# Engineering Single Ni Sites on 3D Cage‐like Cucurbit[n]uril Ligands for Efficient and Selective CO_2_ Photocatalytic Reduction

**DOI:** 10.1002/anie.202417384

**Published:** 2024-11-16

**Authors:** Jingyi Wang, Xiyi Li, Chia‐Hao Chang, Tianyu Zhang, Xuze Guan, Qiong Liu, Liquan Zhang, Ping Wen, Ivan Tang, Yuewen Zhang, Xiaofeng Yang, Junwang Tang, Yang Lan

**Affiliations:** ^1^ Department of Chemical Engineering University College London London WC1E 7JE United Kingdom; ^2^ Centre for Nature-Inspired Engineering University College London London WC1E 7JE United Kingdom; ^3^ Department of Chemistry University College London London WC1H 0AJ United Kingdom; ^4^ College of Environmental Science and Engineering Beijing Forestry University Beijing 100083 China; ^5^ Inorganic Chemistry and Catalysis Group Institute for Sustainable and Circular Chemistry and Debye Institute for Nanomaterials Science Utrecht University Universiteitsweg 99 3584 CG Utrecht, The Netherlands; ^6^ Institute of Analysis Guangdong Academy of Sciences (China National Analytical Center, Guangzhou) Guangzhou 510070 China; ^7^ College of Chemistry and Chemical Engineering Lanzhou University Lanzhou Gansu 730000 China; ^8^ State Key Laboratory of Catalysis Dalian Institute of Chemical Physics Chinese Academy of Sciences Dalian 116023 China; ^9^ Industrial Catalysis Center Department of Chemical Engineering Tsinghua University Beijing 100084 China

**Keywords:** Cucurbit[n]uril, 3D ligand, CO_2_ reduction, molecular co-catalyst, photocatalysis

## Abstract

Solar‐driven CO_2_ selective reduction with high conversion is a challenging task yet holds immense promise for both CO_2_ neutralization and green fuel production. Enhancing CO_2_ adsorption at the catalytic centre can trigger a highly efficient CO_2_ capture‐to‐conversion process. Herein, we introduce cucurbit[n]urils (CB[n]), a new family of molecular ligands, as a key component in the creation of a 3D cage‐like metal (nickel, Ni)‐complex molecular co‐catalyst (CB[7]‐Ni) for photocatalysis. It exhibits an unprecedented CO yield rate of 72.1 μmol ⋅ h^−1^ with a high selectivity of 97.9 % under visible light irradiation. To verify the origin of the carbon source in the products, a straightforward isotopic tracing method is designed based on tandem reactions. The catalytic process commences with photoelectron transfer from Ru(bpy)_3_^2+^ to the Ni^2+^ site, resulting in the reduction of Ni^2+^ to Ni^+^. The locally enriched CO_2_ molecules in the cage ligand CB[7] undergo selective reduction by the Ni^+^ nearby to form CO product. This work exemplifies the inspiring potential of ligand structure engineering in advancing the development of efficient unanchored molecular co‐catalysts.

## Graphical abstract

A novel molecular co‐catalyst (CB[7]‐Ni) is designed with a 3D Cucurbit[7]uril (CB[7]) cage‐like ligand to accommodate atomically dispersed nickel centers for photocatalytic CO_2_ reduction to CO. It achieves a CO yield rate of 72.1 μmol ⋅ h^−1^ with 97.9 % selectivity under visible light irradiation. The homogeneous nature of this co‐catalyst enables efficient charge transfer from photosensitizer to the Ni^2+^ active site, reducing nearby CO_2_ molecules enriched by the unique CB[7] cage.

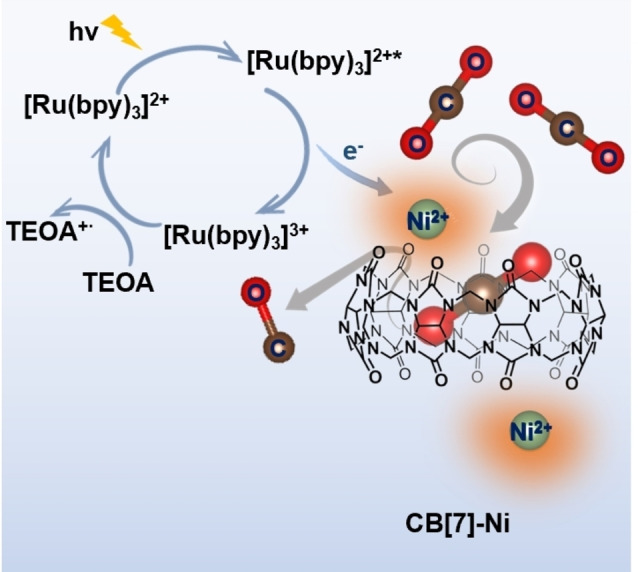

## Introduction

The utilization of carbon dioxide (CO_2_) as a primary substrate for artificial photosynthesis to generate solar fuels and/or valuable chemicals is in line with the growing global demand for carbon neutrality.[^1^, ^2^, ^3^] It presents a promising solution for both mitigating the impacts of greenhouse gas‐induced climate change and reducing reliance on fossil fuels. In general, the CO_2_ photocatalytic reduction process faces challenges of sluggish kinetic rates and poor selectivity in electron utilization. These limitations arise from the involvement of multiple electron transfers (2 e^−^ for CO and HCOOH, 8 e^−^ for CH_4_, etc.) and the competing reaction of proton reduction.[^4^, ^5^, ^6^] Thus, there is a pressing need to develop efficient photocatalysts capable of overcoming these barriers and effectively converting CO_2_ into desired products.

In recent decades, molecular metal‐complex photocatalysts have garnered significant interest owing to their distinct advantages, such as high atomic utilization efficiency, precise control of catalytic sites, high selectivity, low overpotential, and facile detection of intermediate.[^1^, ^7^] To achieve both high yield rate and enhanced selectivity towards desired product, extensive efforts have been devoted to the design and modification of ligands in metal‐complex photocatalysts.

Two‐dimensional (2D) organic molecular ligands, including quaterpyridine,^[8]^ porphyrin,^[6]^ bipyridine,^[9]^ and other N‐heterocyclic compounds,^[10]^ have been extensively investigated due to their complex structures and diverse functional groups capable of accommodating transitional metals. 2D organic molecular ligands allow the manipulation of the electronic properties and the activation configuration of CO_2_ during photocatalytic CO_2_ reduction, thus fine‐tuning the catalytic performance. However, the adsorption of CO_2_ onto catalytic sites is a prerequisite for subsequent catalytic cycles, and 2D organic molecular ligands typically show limited capacity to capture and localize substrate CO_2_ molecules. Consequently, the actual conversion rate of CO_2_ in these catalytic systems often falls below 1 μmol ⋅ h^−1^ (Table S1).

Strategies have been employed to form three‐dimensional (3D) porous structure via decorating molecular catalyst on heterogeneous support^[9]^ or integrating molecular catalysts into frameworks like covalent organic frameworks (COFs) or metal‐organic frameworks (MOFs).^[11]^ However, these structures may suffer from issues such as inhomogeneous distribution, low stability, and inefficient charge transfer between the liquid‐phase photosensitizer and solid‐phase catalytic sites. Moreover, the adsorption sites of CO_2_ are not consistently positioned near the catalytic center, hindering an efficient capture‐to‐convert process.

Therefore, a promising solution is the construction of 3D ligand, which is the direct design of cage‐like organic molecular ligands that maintain a homogeneous nature with 3D porous structures. Compared to the common planar geometries of 2D ligand, the distinct advantage of 3D ligands lies in the confined space (cage) they create, showing the capability to spontaneously encapsulate specific substrate molecules through host–guest interactions.[^12^, ^13^]

**Scheme 1:**
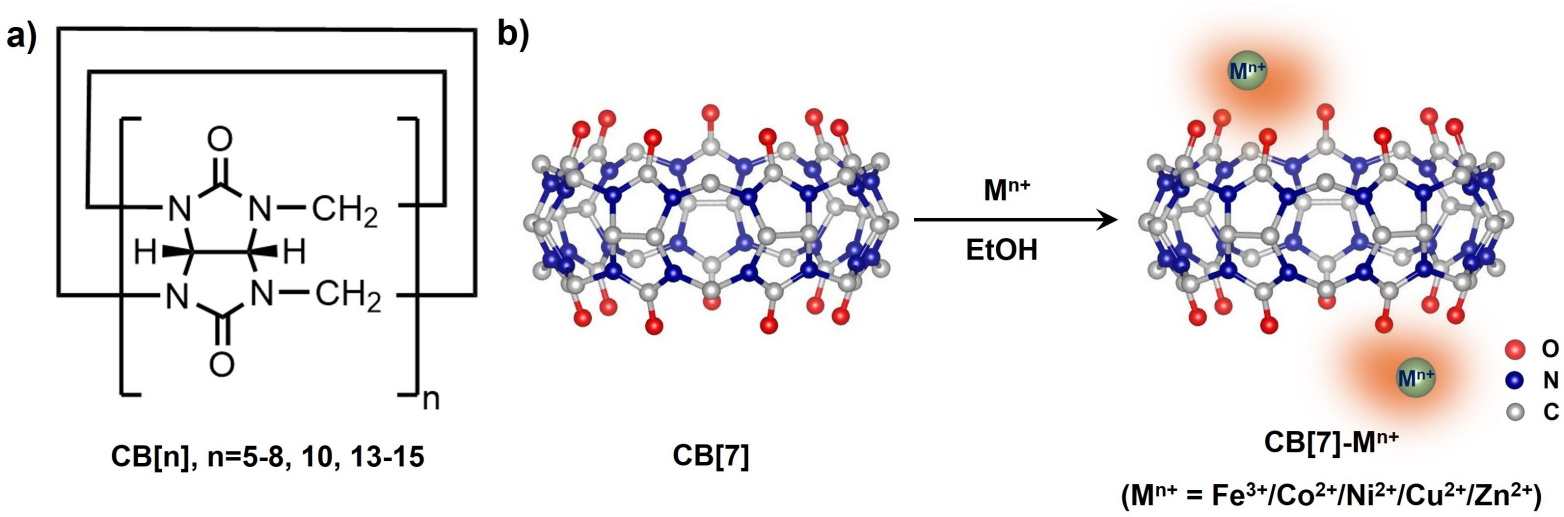
a) Chemical structure of CB[n] (*n*=5–8, 10, 13–15); b) Side view of X‐ray crystallography structures of CB[7]^[23]^ and procedure for the synthesis of CB[7]‐M^n+^.

Cucurbit[n]urils (CB[n], where *n*=5–8, 10, 13–15, as shown in Scheme 1a) are a family of 3D macrocyclic molecules formed by the cyclization of “n” glycoluril units. CB[n] possess remarkable properties for binding guest molecules and coordinating with metal ions.^[13]^ CB[n] molecules consists of a hydrophobic rigid cavity and two identical portals with electron‐rich carbonyl groups.^[14]^ The hydrophobic cavity of CB[n] imparts exceptional gas adsorption capabilities, rivalling those of porous materials such as metal–organic frameworks (MOFs).^[12]^ For example, CB[6] exhibited the ability to trap two CO_2_ molecules within its cavity with a selectivity of 46.4 over CO.^[12]^

Furthermore, the portals of CB[n], with their negatively polarized carbonyl linings, show a strong affinity for coordinating with metal ions.[^15^, ^16^] A systematic and comprehensive understanding on the coordination interactions between CB[n]s and various metal ions has been illustrated by Tao research group.[^17^, ^18^] A few successful examples of CB[n] complexes with different transition metal ions have been developed by them, such as Zn^2+[15]^ and Cd^2+^,^[16]^ which facilitates the development of functional complexes rooted in cucurbit[n]urils. Because of the unique structural properties, recent reports have employed supramolecular self‐assembly strategies to introduce CB[n] to electrocatalysts, such as Au^[19]^ and cuprous oxide (Cu_2_O),^[20]^ to enhance electrocatalytic CO_2_ reduction. In both cases, CB[n] was used as a promoter to modify the nanoparticles. Despite its great potential to work as a proper 3D homogeneous scaffold for engineering single atom catalysts, the development of CB[n]‐metal complex as a state‐of‐the‐art unanchored molecular co‐catalyst for photocatalysis has not been reported to the best of our knowledge.

Herein, a series of transition metal compounds (Fe, Co, Ni, Cu, Zn) coordinated with CB[7] have been investigated for homogeneous photocatalytic CO_2_ reduction. Among these CB[7]‐metal complexes, CB[7]‐Ni complex exhibits exceptional performance, achieving a high CO yield rate of 72.1 μmol ⋅ h^−1^ and a selectivity of 97.9 % under visible light irradiation (λ >420 nm). The CO_2_ conversion rate surpasses that of previously reported photocatalytic homogeneous systems and heterogeneous systems for CO_2_ reduction to CO, underlying the significance of enhancing CO_2_ adsorption through the construction of a 3D organic ligand. In addition, a new and straightforward isotope‐labelling method has been developed through a tandem reaction to rigorously verify the carbon source of the as‐formed CO product in the process.

## Results and Discussion

**Photocatalytic activity**. As shown in Scheme S1, CB[7] is obtained from the separation of CB[n], which is synthesized through the condensation of glycolurils according to the reported procedures (Scheme S1, Figure S1).[^21^, ^22^] Subsequently, widely employed transition metal centers in homogeneous molecular catalysts (Table S1) are introduced separately to coordinate with CB[7], resulting in the formation of CB[7]‐M^n+^ complexes (M ^n+^=Fe ^3+^, Co ^2+^, Ni ^2+^, Cu ^2+^, Zn ^2+^) in an ethanol solution (Scheme 1b).

**Figure 1:**
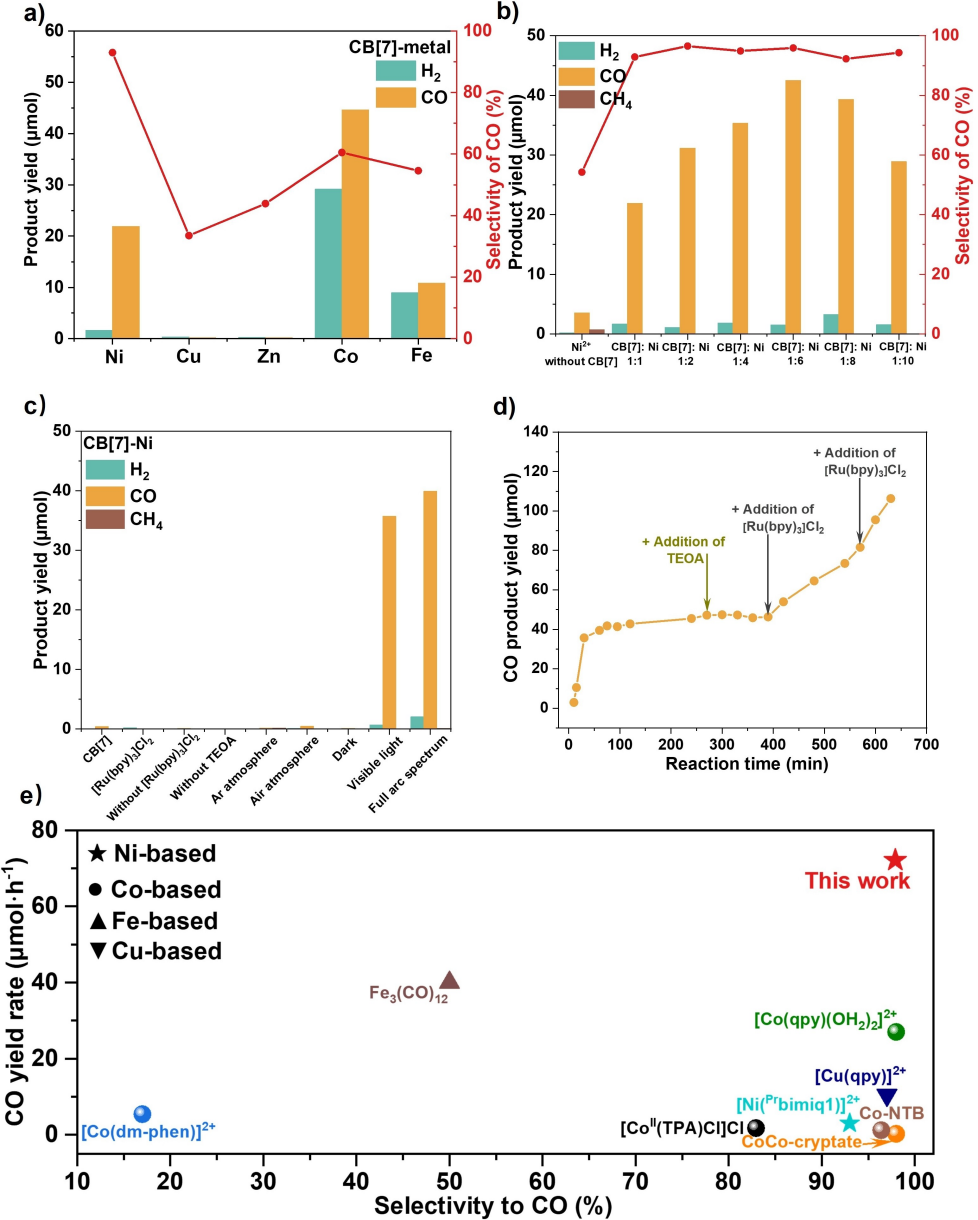
Photocatalytic CO_2_ reduction performances of CB[7]‐M catalysts: a. Product yields and CO selectivity of CB[7]‐M (M=Fe, Co, Ni, Cu, Zn) ; b. Product yields and CO selectivity of CB[7]:Ni synthesized from different ratios of precursors (without CB[7], and CB[7]:Ni=1 : 1, 1 : 2, 1 : 4, 1 : 6, 1 : 8 and 1 : 10); c. Control experiments of CB[7]‐Ni under different conditions (CB[7] without Ni^2+^; [Ru(bpy)_3_]Cl_2_ without CB[7]‐Ni; CB[7]‐Ni without [Ru(bpy)_3_]Cl_2_; without TEOA; under argon atmosphere; under air atmosphere; in dark; under visible light irradiation (λ >420 nm); under full arc irradiation); d. Time course of CO yield on CB[7]‐Ni. (Reaction conditions: 10 mg catalyst, 15 mg [Ru(bpy)_3_]Cl_2_, reaction time 0.5 h, 12 mL solution (CH_3_CN:TEOA:H_2_O=4 : 1 : 1); 300 W Xe lamp of full arc spectrum for Figure a, b and c; 300 W Xe lamp with long‐pass filter (λ >420 nm) for Figure d.) e. Summary of CO selectivity and CO yield rate in this work and other representative counterparts (1. CoCo‐cryptate;^[25]^ 2. [Co^II^(TPA)Cl]Cl;^[26]^ 3. Co‐NTB;^[27]^ 4. [Ni(^Pr^bimiq1)];^[10]^ 5. [Fe_3_(CO)_12_];^[28]^ 6. [Co(qpy)(OH_2_)_2_];^[29]^ 7. [Co(dm‐phen)];^[30]^ 8. [Cu(qpy)]^[31]^)

The photocatalytic CO_2_ reduction potentials of the CB[7]‐M^n+^ complexes are investigated in the presence of a benchmark photosensitizer, Ru(bpy)_3_Cl_2_ (Figure 1a).^[7]^ The CO yield within 30 minutes follows the order of CB[7]‐Co> CB[7]‐Ni> CB[7]‐Fe> CB[7]‐Zn> CB[7]‐Cu. Although CB[7]‐Co exhibits the highest CO production of 44.6 μmol, it simultaneously generates 29.2 μmol H_2_, resulting in a moderate CO selectivity of approximately 60 %. CB[7]‐Ni, on the other hand, shows a CO yield half the amount of CB[7]‐Co but significantly lower H_2_ generation (approximately 1.6 μmol), leading to the highest CO selectivity of 93 %. Considering both the yield rate and selectivity, Ni is selected as the optimal metal for further investigations.

It should be noted that the formation of CB[7]‐Ni involves a dynamic coordination process between CB[7] and Ni(II) ions.[^13^, ^24^] The free Ni(II) ions migrate in the liquid medium, gravitating towards the portals of CB[7] and gradually reaching a coordination equilibrium. Increasing the concentrations of Ni(II) ions can increase the probability of collisions between Ni and CB[7], which can promote a more thorough coordination between CB[7] and Ni(II) ions. Therefore, it is important to investigate the ratios of CB[7] and Ni in the precursor to achieve an ideal structure and decent performance, as shown in Figure 1b. The yield of CO shows a volcano trend with increasing amount of Ni, reaching the highest yield of 42.5 μmol at a precursor (Ni^2+^:CB[7]) ratio of 6 : 1. Excessive loading of Ni has a detrimental effect on the catalytic activity, potentially due to the accumulation of nickel oxide or the blockage of CB[7] cavity. It is worth noting that the Ni counterpart without CB[7] exhibits over ten times lower CO production (only 3.5 μmol) (Figure 1b). This result underscores the indispensable role of CB[7] as a support to stabilize Ni and enrich CO_2_, which will be further substantiated in subsequent sections. Consequently, CB[7]‐Ni obtained using the molar ratio of CB[7]:Ni=1 : 6 in the precursor is selected for further experiments (*Note: the term ‘CB[7]‐Ni’ will specifically refer to the sample synthesized at this ratio unless otherwise specified. The configuration of CB[7]‐Ni is investigated in the latter sections*).

Similarly, CB[6] and CB[8] are also utilized to form CB[n]‐Ni complex molecular co‐catalyst using the same precursor ratio (CB[n]: Ni=1: 6). Despite the different sizes of CB[n], CB[n] all share the key structural properties of the hydrophobic cavities for CO_2_ encapsulation as well as the carbonyl portals with metal ions coordinating tendency. Therefore, similar enhancement of CO yields with high selectivity are observed (Figure S2). These findings suggest the universality of the strategy in constructing 3D ligands to promote CO_2_ reduction over homogeneous photocatalytic systems.

Different catalyst masses, sacrificial agents, and solvents have been investigated (Figure S3–5 with detailed discussion alongside), which shows that catalyst mass of 10 mg with TEOA and MeCN as the sacrificial agent and the solvent can achieve the best performance.

To validate the photocatalytic CO_2_ reduction process, a series of control experiments are conducted (Figure 1c). Almost no activity can be observed when using only Ru(bpy)_3_Cl_2_ or CB[7], indicating that Ni is the exclusive catalytic site for CO_2_ reduction in the reaction. The photocatalytic nature of the reaction has been confirmed as the reaction failed to proceed in the absence of light or photosensitizer. Furthermore, no CO production is observed under an argon (Ar) or air atmosphere, indicating that the carbon source of CO product very likely originates from CO_2_ rather than from photo‐redox organic transformation in the reaction mixture. Further evidence will be provided by the subsequent isotopic measurements.

Moreover, CB[7]‐Ni complex shows only a slightly lower catalytic activity under visible light irradiation compared with that under full arc irradiation, since Ru(bpy)_3_Cl_2_ shows a favorable response in the visible light region, as confirmed by the UV/Vis absorption spectrum (Figure S6). This result highlights a significant potential for solar energy utilization. A time course of CO yield is then monitored under visible light irradiation (Figure 1d). A fast increase in CO yield is observed within 30 minutes. Subsequently, the CO yield gradually reaches a plateau. The addition of the hole scavenger TEOA fails to reinitiate the reaction, while further addition of the photosensitizer Ru(bpy)_3_Cl_2_ partially restores the initial activity. This phenomenon is commonly observed in reaction systems using Ru(bpy)_3_Cl_2_ as a photosensitizer.^[7]^ Therefore, the disappearance of photocatalytic CO_2_ reduction is likely due to the degradation of the photosensitizer.

To prove the universality of CB[7]‐Ni to work with other photosensitizers, an organic and common dye Rhodamine B (RhB) is used to test the CO_2_ reduction activity (Table S3). The high selectivity of CO (81.4 %) is still maintained, suggesting the potential of CB[7]‐Ni to be coupled with low‐cost and simple photosensitizers for future application. The lower yield rate of CO using RhB as photosensitizer is likely due to the lower reduction capacity of RhB than the noble metal complex Ru(bpy)_3_Cl_2_.^[32]^

To confirm the reproducibility of this molecular co‐catalyst, three CB[7]‐Ni samples are synthesized at different batches and tested under visible light irradiation (Figure S7). The CO yield rate and CO selectivity exhibit similar values across the three different samples, suggesting the high reproductivity of this facile coordination method for constructing CB[7]‐Ni with a 3D ligand. The average CO yield rate and CO selectivity are 72.1 μmol ⋅ h^−1^ and 97.9 %, respectively, under visible irradiation. No other gas products (e.g., CH_4_, C_2_H_6_) or liquid products (e.g., HCOOH, CH_3_OH, etc.) are observed (Figure S8). To the best of our knowledge, this represents the highest yield rate with a decent selectivity among the reported homogeneous photocatalytic systems for CO_2_ reduction to CO (Figure 1e, Table S1). Furthermore, this activity also outperforms the reported heterogeneous photocatalysts (Table S2), particularly considering that the CO yield rate is roughly seven to hundreds of times higher than that of heterogeneous photocatalysts with comparable CO selectivity.

For a comprehensive comparison of performance metrics, CB[7]‐Ni also exhibits a high TON of 1844 and TOF of 1.0 s^−1^, which represents an advanced level compared to the reported homogeneous photocatalytic systems (Table S1). Unlike homogeneous photocatalytic systems, TON/TOF are less reported in heterogeneous photocatalytic systems (Table S2), but CB[7]‐Ni still at the leading position for the CO yield rate using the unit of “μmol g^−1^_cat_ h^−1^”.

The quantum yield of this photocatalytic system is determined to be approximately 1.34 % (λ=450 nm) (detailed calculation shown in the Supporting Information). For those systems with high selectivity to CO, the quantum yield of this work is higher than most of homogeneous/heterogeneous photocatalytic systems (Table S1 and S2). This indicates the high visible light utilization efficiency of this work.

The isotope labelling experiment using ^13^CO_2_ as the feedstock is an essential and widely used tool to confirm the carbon source of the products in photocatalytic CO_2_ reduction reactions. However, conducting such experiments can be challenging when CO is produced as the main product because ^13^CO_2_ (m/z=45) can always generate the molecular fragment of ^13^CO (m/z=29) in the detector of mass spectroscopy (MS). Unless an appropriate separation set‐up, such as GC with the correct column, is used to fully separate CO and CO_2_ before entering the MS ion box, the MS results are invalid and may provide incorrect information. However, we have observed that most of reported GC‐MS machines lack the appropriate column to achieve this separation, resulting in the simultaneous detection of m/z of 29 and 45. Therefore, we have developed a new method to identify the carbon source via tandem reaction combined with either ^13^C NMR or MS analysis.

**Scheme 2:**
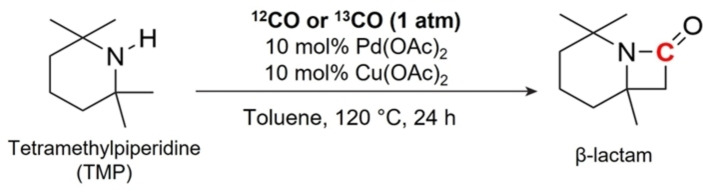
Reaction of intramolecular carbonylation catalysis.

In this approach, a straightforward intramolecular carbonylation reaction is employed to synthesize β‐lactam by reacting tetramethylpiperidine (TMP) with the as‐produced CO (Scheme 2).^[33]^ Since CO_2_ cannot participate this reaction, interference from CO_2_ can be avoided during the analysis of β‐lactam. As shown in Figure S9, the chemical shift at approximately 165.6 ppm in the ^13^C NMR spectrum is assigned to the carbonyl group formed by the insertion of as‐produced CO. Interestingly, using ^13^CO_2_ as the feedstock results in approximately 20‐fold higher intensity compared to using ^12^CO_2_ (*note: even* ^*12*^ *CO_2_ gas can contain trace amounts of* ^*13*^ *CO_2_ in nature*), providing evidence that CO_2_ serves as the sole carbon source. This conclusion is further consolidated by the MS detection of β‐lactam (Figure S10). The molecular weight [M+H^+^] of 169 is obtained under ^13^CO_2_ reaction atmosphere, which is exactly 1 mass number higher than that obtained under ^12^CO_2_ reaction atmosphere. To double confirm the CO product only from CO_2_ rather than other organic species, the gas product CO from ^13^CO_2_ isotope labelling experiment is further analyzed by a GC‐MS with proper separation configuration. As shown in the mass spectrum (Figure S11), only molecular fragment m/z of 29 can be observed. No m/z of 28 assigned to ^12^CO can be found. Thus, the origin source of CO is solely from CO_2_.

**Structure characterization**. The composition, structure and chemical status of CB[7]‐Ni are thoroughly investigated. Upon the introduction of Ni species to CB[7], two additional broad peaks appear in the UV/Vis diffuse reflectance spectroscopy (DRS) spectrum (Figure S12). The first broad peak at around 360–450 nm is attributed to ligand‐to‐metal charge transfer (LMCT) between CB[7] and Ni, while the second absorption band at around 600–800 nm is owing to the d‐d transition of Ni^2+^.^[34]^ The concentration of Ni is determined to be 2.7 wt % by ICP‐OES (Table S4), but no new peak assigned to Ni species can be observed in the X‐ray diffraction (XRD) pattern of CB[7]‐Ni (Figure S13). This observation suggests that Ni ions are uniformly distributed rather than aggregated after coordination with CB[7].^[35]^

**Figure 2:**
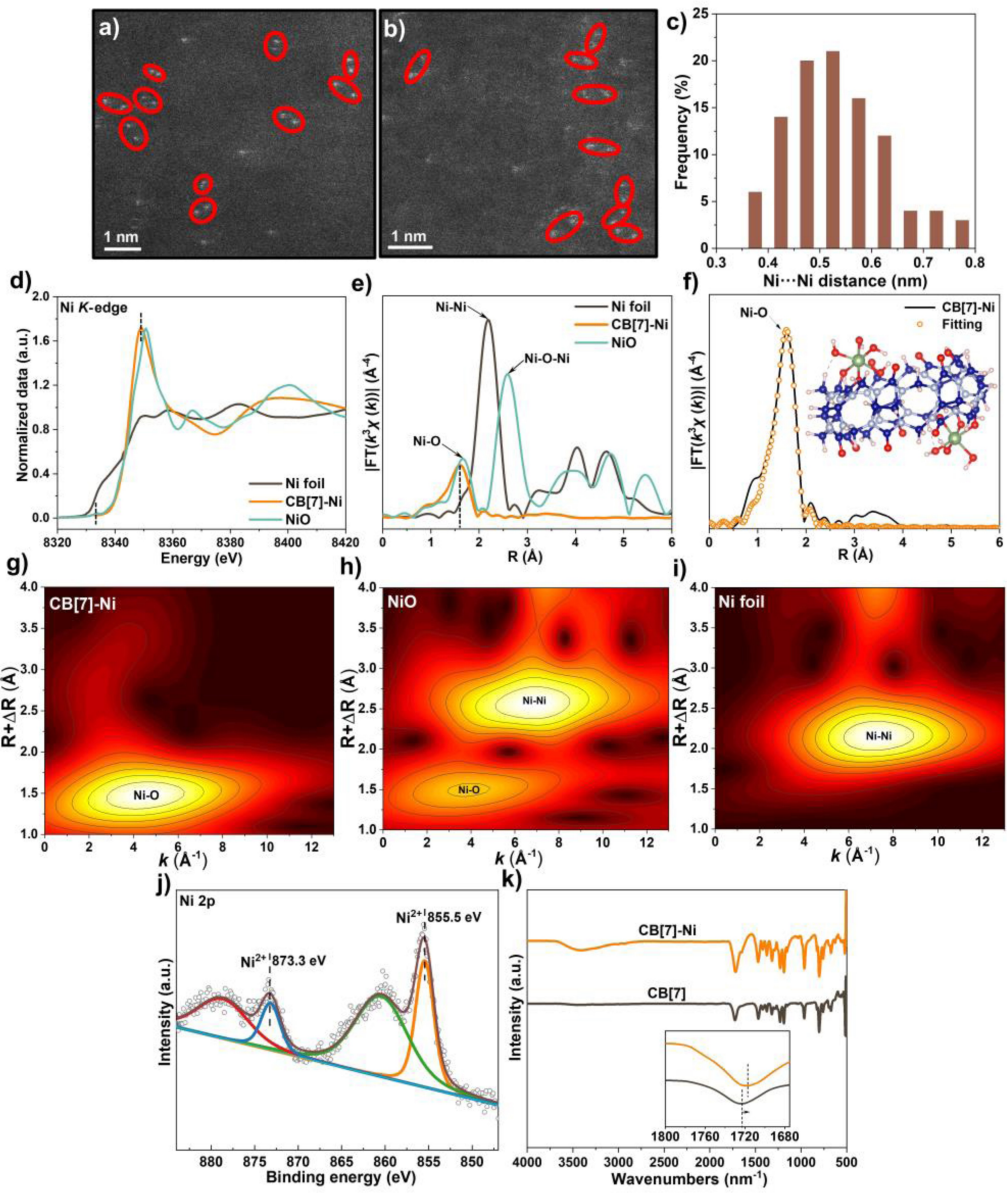
a‐b. Aberration‐corrected HAADF‐STEM images of CB[7]‐Ni at different representative areas; c. Statistical distribution of the Ni⋅⋅⋅Ni distance from STEM images; d. Normalized Ni K‐edge XANES spectra of CB[7]‐Ni, standard Ni foil and NiO; e. *k* ^2^‐weighted Fourier‐transform of the Ni K‐edge EXAFS spectra for CB[7]‐Ni, standard Ni foil and NiO; f. Experimental and fitted EXAFS data of CB[7]‐Ni at the R space (inset, the model of CB[7]‐Ni); g‐i. Wavelet transformed EXAFS spectra of CB[7]‐Ni, standard NiO and Ni foil; j. Ni2p high‐resolution XPS spectrum of CB[7]‐Ni; k. FT‐IR spectra of CB[7] and CB[7]‐Ni (the inset is the zoom‐in of spectra from 1800–1675 cm^−1^).

The atomic structure of the Ni species on CB[7] is further revealed by using aberration correction high angle annular dark field scanning transmission electron microscopy (HAADF‐STEM) imaging (Figure 2a–b). As expected, bright spots with higher contrasts compared to the substrate are observed in adjacent and paired form (highlighted by red circles to emphasize those representatives without multi‐molecules overlap during STEM observation). These small paired bright dots are ascribed to the atomically dispersed Ni located at the two portals of each CB[7] molecule, considering their higher contrast (M=58.69 for Ni) than CB[7] (M=12.0, 14.0, 15.99 for C, N, O). The statistical analysis of 100 pairs of bright dots displays a wide range of Ni⋅⋅⋅Ni distance, from approximately 0.4–0.8 nm (Figure 2c).

X‐ray absorption spectroscopy (XAS) is carried out to gain insights into the chemical status and localized coordination environment of Ni in the CB[7]‐Ni complex. The pre‐edge absorption and white line features of CB[7]‐Ni closely resemble those of NiO and are higher than those of Ni foil, indicating the presence of a divalent state of Ni species (Figure 2d).^[36]^ This observation is further supported by X‐ray photoelectron spectroscopy (XPS) analysis (Figure 2j), which reveals binding energy of Ni 2p_3/2_ and Ni 2p_1/2_ is at 855.5 and 873.3 eV, respectively, corresponding to the Ni^2+^ chemical state.^[37]^ The k^2^‐weighted Ni K‐edge extended X‐ray absorption fine structure (EXAFS) spectra of CB[7]‐Ni, Ni foil and NiO are compared in Figure 2e. CB[7]‐Ni exhibits first‐shell scattering at approximately 1.6 Å in R space, which is in close proximity to that in NiO. The absence of Ni−Ni peaks and Ni−Ni O−Ni peaks further supports the atomic dispersion of Ni with CB[n], consistent with the findings from the HAADF‐STEM imaging. The corresponding Ni K‐edge EXAFS fitting of CB[7]‐Ni reveals a Ni−O coordination number of 6.3±0.6, with a bond length of approximately 2.0 Å (Figure 2f, Table S5).

To resolve the coordination structure of Ni, the DFT simulation is performed as shown in Figure S14 with detailed discussion alongside. Considering the Ni is probably a hexacoordinated octahedral structure based on the EXAFS result, aside from the coordination of one Ni with two oxygen atoms from the CB[7] ligand, the remaining oxygen coordination is likely from ‐OH groups. This hypothesis is corroborated by the emergence of a new broad absorption band, indicative of ‐OH stretching vibration, at approximately 3450 cm^−1^ in the Fourier transformation infrared spectroscopy (FTIR) (Figure 2k).^[38]^ Then, DFT simulation is further performed using OH groups to compensate the rest coordination sites. After calculation optimizations, a hexacoordinated octahedral structure for Ni was established (the inset of Figure 2f). To further verify this structure, this model is used to fit the EXAFS data again and the corresponding new fitting curve was shown in Figure S15. The fitting curve of the DFT‐optimized structure matches well with the experimental data, confirming the validity of the fitted hexacoordinated octahedral structure for CB[7]‐Ni. Compared to standard NiO and Ni foil, the wavelet‐transformed EXAFS spectrum of CB[7]‐Ni (Figure 2g) only exhibits a maximum intensity center corresponding to Ni−O interaction, without the presence of a Ni−Ni interaction, which is different from that of NiO (Figure 2h) and Ni foil (Figure 2i). This further confirms the isolation and dispersion of Ni atoms on CB[7] ligand.

The complexation of Ni and CB[7] is also confirmed by FTIR, as shown in Figure 2k. The main absorption peaks corresponding to CB[7] are assigned and presented in Table S6.^[39]^ The FTIR spectrum of CB[7]‐Ni is similar to that of CB[7], indicating the preserved structure of CB[7] after the complexation process. Importantly, the peak attributed to carbonyl groups of CB[7]‐Ni shows a red shift of approximately 10 cm^−1^ compared to that in CB[7] (the inset of Figure 2k). This shift is attributed to the strong interaction between Ni^2+^ and the carbonyl group in CB[7] from the chelation.[^35^, ^40^] Such chelation interaction is further studied by ^1^H NMR as shown in Figure S16. Upon the addition of Ni species, significant line broadening of the resonance is observed. Moreover, two double peaks H_a_ and H_b_ from the diastereotopic CH_2_‐ group merge into a single peak and shift downfield. These changes in the ^1^H NMR demonstrate the binding effect between Ni and CB[7],^[41]^ thereby supporting the findings from EXAFS and FTIR analyses.

**Figure 3:**
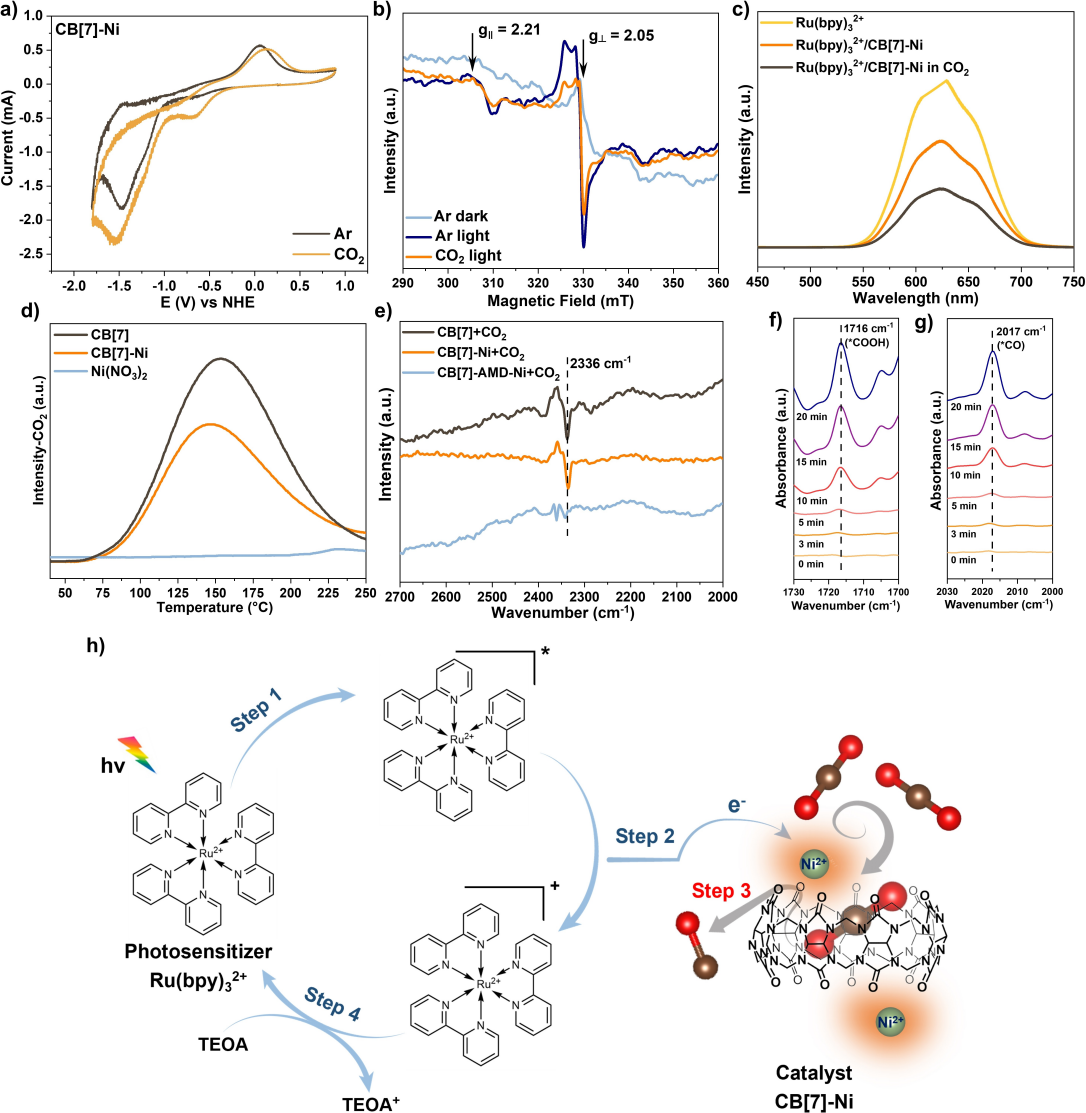
a. Cyclic voltammogram of CB[7]‐Ni under Ar atmosphere and CO_2_ atmosphere in 0.1 M NBu_4_PF_6_/DMF solution (pH=9.9) with scan rate at 100 mV ⋅ s^−1^; b. In situ EPR spectra of CB[7]‐Ni with Ru(bpy)_3_Cl_2_ in CH_3_CN under Ar atmosphere in the dark condition and under light irradiation, as well as in CO_2_ atmosphere under light irradiation at 77 K; c. In situ steady‐state PL spectra of [Ru(bpy)_3_]Cl_2_ solution (10 mL CH_3_CN + 2 mL H_2_O), CB[7]‐Ni mixed [Ru(bpy)_3_]Cl_2_ solution and CB[7]‐Ni mixed [Ru(bpy)_3_]Cl_2_ solution under CO_2_ atmosphere; d. TPD‐CO_2_ profiles of CB[7], CB[7]‐Ni and Ni(NO_3_)_2_; e. ATR‐FTIR spectra of CB[7], CB[7]‐Ni and CB[7]‐AMD−Ni under CO_2_ atmosphere; f. and g. In situ ATR‐FTIR spectra of CB[7]‐Ni at different reaction times (Reaction condition: 10 mg CB[7]‐Ni; 15 mg [Ru(bpy)_3_]Cl_2_; 12 mL mixed solvent (CH_3_CN: H_2_O=5 : 1); 300 W Xe lamp (λ >420 nm)); h. Schematic process for photocatalytic CO_2_‐to‐CO conversion based on [Ru(bpy)_3_]Cl_2_ as the photosensitizer and CB[7]‐Ni as the catalyst.

**Charge transfer pathway**. The mechanism of photocatalytic CO_2_ reduction over CB[7]‐Ni is then investigated. To gain more insights into the catalytic site for CO_2_ reduction, the cyclic voltammetry (CV) of CB[7]‐Ni and CB[7] under either Ar or CO_2_ atmosphere is carried out as depicted in Figure 3a and Figure S17. Under Ar atmosphere, the CV of CB[7] shows a single reduction peak at approximately −0.59 V vs. NHE, whereas CB[7]‐Ni displays two reduction peaks at around −0.59 V and −1.50 V vs. NHE. The reduction peak at −0.59 V vs. NHE is assigned to the proton reduction, which might be from moisture absorption in the hydroscopic DMF solution (detailed discussion shown alongside Figure S17).^[42]^ The reduction peak observed at −1.50 V vs. NHE over CB[7]‐Ni can be attributed to the reduction of Ni(II)/Ni(I) species, in line with the previous studies.[^43^, ^44^] An enhanced current, indicative of electrochemical CO_2_ reduction, is observed at −1.50 V vs. NHE for CB[7]‐Ni, suggesting that the as‐formed reduction species Ni^+^ is capable of triggering CO_2_ reduction.^[8]^ In contrast, negligible current enhancement is observed over CB[7] under CO_2_ atmosphere, suggesting the absence of an active site for CO_2_ reduction on CB[7] (Figure S17). This result is also in agreement with the photocatalytic CO_2_ reduction activity observed in Figure 1c. The reduction potential of Ru(bpy)_3_Cl_2_ could reach −1.52 V vs. NHE,[^45^, ^46^] suggesting that the excited electron from Ru(bpy)_3_Cl_2_ can be transferred to the Ni^2+^ site on CB[7]‐Ni for CO_2_ reduction under light irradiation. The DFT simulation also verifies the charge transfer from Ru(bpy)_3_Cl_2_ to Ni^2+^ (detailed discussion shown alongside Figure S18).

To provide direct evidence of charge transfer at the Ni^2+^ catalytic center, the in situ electronic paramagnetic resonance (EPR) measurements are performed under various reaction conditions at 77 K (Figure 3b). It should be noted that acquiring a high‐quality signal of Ni^+^ is challenging due to its highly active nature and the requirement of low temperature such as 77 K using liquid nitrogen.^[36]^ During the experiments, Ru(bpy)_3_Cl_2_ and CB[7]‐Ni are dispersed in CH_3_CN and Ar or CO_2_ is used to create the gas atmosphere in the sealed tubes. Under light irradiation, the EPR spectrum shows significant changes compared to that under dark conditions. The characteristic of Ni^+^ (S=1/2) can be identified, which displays the g‐factor anisotropy (g_‖_=2.21, g_⊥_=2.05) due to the axial symmetry of the first coordination sphere of CB[7]‐Ni.^[47]^ Despite some noise, a partially resolved hyperfine splitting of A_⊥_=1.05 mT from oxygen atoms can be observed. This experiment provides evidence that the Ni^2+^ in CB[7]‐Ni can accept the photoelectrons from Ru(bpy)_3_Cl_2_ under light irradiation. Next, CO_2_ is introduced to the system, and the intensity of the Ni^+^ signal exhibits a decrease, suggesting charge transfer from Ni^+^ to CO_2_. In other words, the CO_2_ can be reduced by Ni^+^, which is consistent with the electrochemical measurement.

To further verify the charge transfer process, in situ photoluminescence (PL) measurements are conducted under different conditions (Figure 3c). Excited by 325 nm laser, the Ru(bpy)_3_Cl_2_ solution shows a strong emission in the range of 550–700 nm under Ar atmosphere. The introduction of CB[7]‐Ni to the solution, however, causes a significant reduction in the emission intensity, suggesting the photoelectron transfer from Ru(bpy)_3_Cl_2_ to CB[7]‐Ni. Subsequently, purging CO_2_ to replace Ar, the emission intensity exhibits an obvious further decline. This finding supports the notion that the photoelectron can transfer to CB[7]‐Ni and subsequently reduce CO_2_, which corroborates the analyses from the CV and in situ EPR results. Therefore, under light irradiation, the ground state Ru(bpy)_3_^2+^ absorbs photons, leading to photoinduced electrons and holes at its LUMO and HOMO, respectively. Through intersystem crossing, the excited singlet state Ru(bpy)_3_^2+^* transitions to a triplet state with a longer lifetime and higher reducing potential.[^48^, ^49^] This triplet state can transfer an electron to Ni^2+^, generating the oxidised Ru(bpy)_3_^3+^ and the reduced Ni^+^. Subsequently, CO_2_, selectively enriched by the cage ligand CB[7], is reduced by nearby Ni^+^ species to form CO molecules with remarkably high selectivity. In the oxidative quenching process of the photosensitizer, TEOA, as an electron donor, provides electrons to Ru(bpy)_3_^3+^, restoring it to its original state Ru(bpy)_3_^2+^. This completes the photoreduction process of Ru(bpy)_3_^2+^ and prepares it to initiate the next cycle of light absorption, excitation, and electron transfer.

**Reaction mechanism**. After the investigation of the charge transfer and the CO_2_ reduction performance, the role of CB[7] in enhancing CO_2_ capture is explored. Temperature programmed CO_2_ desorption (TPD‐CO_2_) experiments are conducted to investigate the adsorption of CO_2_ over CB[7], CB[7]‐Ni and Ni species without CB[7] modification (Figure 3d). The pristine CB[7] exhibits a strong CO_2_ adsorption capacity, as evidenced by a desorption peak at approximately 153 °C with high intensity. Interestingly, CB[7]‐Ni also displays a desorption peak at around 146 °C, albeit with a slight decrease in intensity. This suggests that the majority of CO_2_ adsorption capacity is retained even after coordination with Ni. In contrast, Ni(NO_3_)_2_, which represents the Ni species without CB[7] modification, shows no CO_2_ adsorption capacity.

CO_2_ adsorption isotherms are further conducted to investigate the CO_2_ adsorption capacity (Figure S19). The pure Ni species without CB[7] modification exhibits no CO_2_ adsorption capability. In comparison, the CB[7]‐Ni shows a strong adsorption capacity (1.43 mmol ⋅ g^−1^), which is slightly lower than that of CB[7] (1.84 mmol ⋅ g^−1^). This verifies that CB[7]‐Ni retains most of the inherent CO_2_ adsorption capability of CB[7], which is also consistent with the findings from TPD‐CO_2_ measurements. In addition, comparing the electrostatic potential maps of CB[7] and CB[7]‐Ni as shown in Figure S20, a slight change in charge states of CB[7] after coordination with Ni indicates that the enhancement of CO_2_ adsorption is attributed to the CB[7] cage itself rather than the changed charge states from Ni atom. All results imply that the coordination of Ni at the portals of CB[7] does not hinder CO_2_ adsorption by the CB[7] cavity, likely due to the dynamic coordination nature as previously discussed.

Furthermore, the temperature programmed H_2_O desorption (TPD‐H_2_O) over CB[7]‐Ni is conducted by thermogravimetric analysis‐mass spectrometry (TGA‐MS), as shown in Figure S21. The primary H_2_O desorption peak in CB[7]‐Ni occurs at approximately 100 °C, which is about 50 °C lower than the temperature of CO_2_ desorption peak on CB[7]‐Ni. This indicates that CB[7]‐Ni exhibits a stronger interaction with CO_2_ compared to H_2_O due to the hydrophobic nature of CB[7] cavity.^[13]^ This property likely contributes to the unique high photocatalytic selectivity of CB[7]‐Ni for CO_2_ reduction to CO rather than reducing H_2_O to H_2_. DFT simulation is further used to compare the potential energy barriers of hydrogen evolution reaction during CO_2_ reduction on CB[7]‐Ni, as shown in Figure S22. The competing proton reduction has a barrier of 0.87 eV, which is higher than the 0.60 eV barrier for CO formation in photocatalytic CO_2_ reduction. This result indicates the effective suppression of the hydrogen evolution reaction on CB[7]‐Ni in the experiment.

To verify the CO_2_ adsorption site in CB[7]‐Ni, a guest replacement experiment is designed using FTIR for detection. As shown in Figure 3e, a peak at 2336 cm^−1^ is detected in the FTIR spectrum of CB[7] after CO_2_ adsorption, which is assigned to the asymmetric stretching mode of CO_2_ confined within the CB[n] cavity.[^14^, ^50^] Importantly, a similar peak is observed in CB[7]‐Ni after CO_2_ adsorption, suggesting the maintenance of the CO_2_ adsorption capacity with the presence of Ni at the portals of CB[7]. This is reasonable since Ni is chelated at the portal of CB[7] rather than aggregating within the CB[7] cavity. Moreover, the coordination between Ni and carbonyl groups at the portal of CB[7] is a dynamic process.^[14]^ To confirm this assignment, amantadine (AMD), a well‐known CB[7] guest molecule with a strong binding affinity to CB[7], is added to the cavity of CB[7] (successful encapsulation is confirmed by ^1^H NMR in Figure S23).[^51^, ^52^] The FTIR spectrum is then measured again, and the peak at 2336 cm^−1^ disappears, indicating the competitive displacement of CO_2_ from the cavity by AMD. These findings indicate that the CB[7] cavity can exert CO_2_ enrichment function in CB[7]‐Ni during the photocatalytic CO_2_ reduction.

To confirm this CO_2_ enrichment function, the 2‐dimensional molecule glycoluril (basic structural unit of CB[7]) as the 2D ligand for Ni complexation is used and tested for photocatalytic CO_2_ reduction. To achieve a consistent molar concentration of carbonyl groups in the reaction system, the control experiment maintained a molar ratio of glycolurils to Ni^2+^ at 7 : 2, considering seven glycoluril units in one CB[7] structure with two Ni^2+^ ions at the portals. The performance of photocatalytic CO_2_ reduction in glycolurils‐Ni is presented in Figure S24. It should be noted that the CO yield rate of CB[7]‐Ni is approximately 3.2 times higher than that of glycolurils‐Ni. Therefore, the formation of the 3D cage ligand structure has a more pronounced effect than the simple coordination interaction, which enhances the adsorption of CO_2_ to provide more substrate for conversion at the local environment.

To further demonstrate the important contribution of CO_2_ enrichment by the cavity of CB[7] during the photocatalytic CO_2_ reduction, an elaborate control experiment is designed, as shown in Figure S25. Both CB[7]‐Ni and CB[7]‐AMD−Ni solid samples are exposed to CO_2_ gas for 2 hours, after which they are transferred to the photocatalytic reaction solution, respectively. The reaction system is then purged with Ar to create an inert atmosphere prior to the photocatalytic reaction. By conducting this experiment, the over‐saturated pure CO_2_ atmosphere in the typical photocatalytic CO_2_ reduction system can be avoided, allowing for a scientific evaluation of the function of CB[7] in capturing CO_2_. It is anticipated that CB[7]‐Ni, with its empty CB[7] cavity, would adsorb more CO_2_ during the CO_2_ flow for subsequent photocatalytic reduction compared to CB[7]‐AMD−Ni (the cavity of CB[7] is preoccupied by AMD). The CO yield over CB[7]‐Ni is around two times than that of CB[7]‐AMD−Ni, thus confirming the unique function of CB[7] in enriching CO_2_ molecules for photocatalytic reduction.

DFT simulation is further used to obtain a more comprehensive understanding of the CO_2_ reduction pathway on the CB[7]‐Ni. The adsorption of CO_2_ on CB[7]‐Ni has been considered first (Figure S26). It should be noted that the adsorption energy of CO_2_ in the cage of CB[7] is 0.20 eV lower than that on the surface of CB[7], indicating that the encapsulation of CO_2_ in the cage of CB[7] is more stable than on the surface. Thus, the cage of CB[7] can facilitate the CO_2_ adsorption to enrich more CO_2_ to participate the CO_2_ reduction on the Ni site, which is consistent with the experimental results above. Then, the adsorption of CO_2_ in the cage of CB[7]‐Ni was used as the initial state for the following CO_2_ reduction reaction (Figure S27). The incorporation of a proton (H^+^) and a photoelectron can lead to the formation of CO‐OH intermediate on the Ni site, surmounting an energy barrier of 0.60 eV. Then, the additional integration of another proton and photoelectron facilitates the dissociation of the C−O bond, resulting in the production of CO and H_2_O. Apart from the activation of CO_2_ to form CO‐OH, the rest pathway is thermodynamically favorable, which is likely to be another reason for the high performance of this catalyst.

In situ ATR‐FTIR spectra were further performed to verify the reaction intermediates in the CO_2_ photoreduction process, as shown in Figure 3f and 3 g. Upon illumination, the characteristic absorption bands corresponding to the *COOH and *CO intermediates appear at 1716 and 2017 cm^−1^, respectively.[^53^, ^54^] Meanwhile, the intensity of these absorption peaks gradually increases with illumination time. The presence of these species confirms the formation of *COOH and *CO intermediates during the CO_2_ photoreduction process.

To obtain a more comprehensive understanding of the CO_2_ reduction pathway on CB[7]‐Ni, other possible reaction pathways leading to CH_4_ or C_2_H_4_ products were also calculated as shown in Figure S28 with discussions alongside. The comparison of the energy barriers across the three catalytic pathways highlights the favorable pathway for CO_2_‐to‐CO conversion, consistent with the high CO yield observed for CB[7]‐Ni in experimental performance results.

Based on the aforementioned results, a Scheme for the photocatalytic CO_2_ reduction over CB[7]‐Ni is proposed (Figure 3h). Under light irradiation, the excited photoelectron on Ru(bpy)_3_Cl_2_ can transfer to the Ni^2+^ site in CB[7]‐Ni, reducing Ni^2+^ to Ni^+^. Subsequently, CO_2_, selectively enriched by the 3D organic cage ligand CB[7], is reduced by nearby Ni^+^ species to form CO molecules with remarkably high selectivity.

## Conclusions

In this study, a novel unanchored molecular co‐catalyst, CB[7]‐Ni, featuring a 3D cage‐like ligand, has been developed for the first time and its photocatalytic CO_2_ reduction activity has been evaluated. By optimizing the ratio between CB[7] and Ni, a structure with two Ni atoms located at the two portals of CB[7] can be obtained. Benefiting from the selective enrichment of CO_2_ by the CB[7] cage, CB[7]‐Ni exhibits the highest CO yield rate of 72.1 μmol ⋅ h^−1^, with a selectivity of 97.9 % under visible light irradiation, among the reported photocatalysts in CO_2_‐to‐CO conversion processes. In the meantime, a new rigorous isotopic tracing method has been developed via intramolecular carbonylation tandem reaction, solidly confirming CO_2_ as the single carbon source for CO product. Various characterization experiments, including CV, in situ EPR, and in situ PL confirm that the Ni^2+^ on CB[7] is the active center that accepts photoelectron from Ru(bpy)_3_Cl_2_ to form Ni^+^. The resulting Ni^+^ species then reduce the nearby CO_2_ molecules captured by the cage of CB[7]. The homogeneous nature of CB[7]‐Ni allows efficient charge transfer between the photosensitizer and the active center, while enhancing CO_2_ adsorption in the hydrophobic cage of CB[7] to promote the catalytic reaction. Overall, this work presents a new perspective on ligand structure engineering in homogeneous molecular co‐catalyst, offering insights for improving their catalytic activity and selectivity.

## Author Contributions

Y. L. and J. T. conceived and supervised the progress of the entire project. X. L. conceived the experiments, data analysis, mechanism investigation and discussion. J. W. conducted the catalysts preparation, performance tests and major sample characterizations. C. C. contributed to tandem reaction and analysis of isotope labelling products. T. Z. and X. Y. carried out the DFT simulations. X. G. performed EXAFS fitting and provided the related discussion. Q. L. and I. T. carried out the TPD‐CO_2_ experiments and discussed the results. P.W. and W.Z. conducted the HAADF‐STEM imaging and analysis. L. Z. assisted with the electrochemical tests.

## Conflict of Interests

The author declares no conflicts of interest.

1

## Supporting information

As a service to our authors and readers, this journal provides supporting information supplied by the authors. Such materials are peer reviewed and may be re‐organized for online delivery, but are not copy‐edited or typeset. Technical support issues arising from supporting information (other than missing files) should be addressed to the authors.

Supporting Information

## Data Availability

The data that support the findings of this study are available from the corresponding author upon reasonable request.

## References

[1] L. Chen , G. Chen , C.-F. Leung , C. Cometto , M. Robert , T.-C. Lau , Chem. Soc. Rev. 2020, 49, 7271–7283. 32954394 10.1039/d0cs00927j

[2] Z. Wang , H. Song , H. Liu , J. Ye , Angew. Chem. Int. Ed. 2020, 59, 8016–8035. 10.1002/anie.201907443 31309678

[3] H. Wu , X. Li , C. Tung , L. Wu , Adv. Mater. 2019, 1900709. 10.1002/adma.201900709 31271262

[4] H. Rao , C. H. Lim , J. Bonin , G. M. Miyake , M. Robert , J. Am. Chem. Soc. 2018, 140, 17830–17834. 30525556 10.1021/jacs.8b09740 PMC6467819

[5] H. Rao , L. C. Schmidt , J. Bonin , M. Robert , Nature 2017, 548, 74–77. 28723895 10.1038/nature23016

[6] J. Bonin , M. Robert , M. Routier , J. Am. Chem. Soc. 2014, 136, 16768–16771. 25396278 10.1021/ja510290t

[7] J.-W. Wang , F. Ma , T. Jin , P. He , Z.-M. Luo , S. Kupfer , M. Karnahl , F. Zhao , Z. Xu , T. Jin , T. Lian , Y.-L. Huang , L. Jiang , L.-Z. Fu , G. Ouyang , X.-Y. Yi , J. Am. Chem. Soc. 2023, 145,676–688. 36538810 10.1021/jacs.2c11740

[8] Y. Wei , L. Chen , H. Chen , L. Cai , G. Tan , Y. Qiu , Q. Xiang , G. Chen , T. C. Lau , M. Robert , Angew. Chem. Int. Ed. 2022, 61, e202116832. 10.1002/anie.202116832 34986281

[9] J. Lin , Y. Hou , Y. Zheng , X. Wang , Chem. Asian J. 2014, 9, 2468–2474. 24986767 10.1002/asia.201402303

[10] V. S. Thoi , N. Kornienko , C. G. Margarit , P. Yang , C. J. Chang , J. Am. Chem. Soc. 2013, 135, 14413–14424. 24033186 10.1021/ja4074003

[11] H. Zhang , J. Wei , J. Dong , G. Liu , L. Shi , P. An , G. Zhao , J. Kong , X. Wang , X. Meng , J. Zhang , J. Ye , Angew. Chem. Int. Ed. 2016, 128, 14522–14526.

[12] H. Kim , Y. Kim , M. Yoon , S. Lim , S. M. Park , G. Seo , K. Kim , J. Am. Chem. Soc. 2010, 132, 12200–12202. 20718409 10.1021/ja105211w

[13] S. J. Barrow , S. Kasera , M. J. Rowland , J. Del Barrio , O. A. Scherman , Chem. Rev. 2015, 115, 12320–12406. 26566008 10.1021/acs.chemrev.5b00341

[14] A. Wagner , K. H. Ly , N. Heidary , I. Szabó , T. Földes , K. I. Assaf , S. J. Barrow , K. Sokołowski , M. Al-Hada , N. Kornienko , M. F. Kuehnel , E. Rosta , I. Zebger , W. M. Nau , O. A. Scherman , E. Reisner , ACS Catal. 2020, 10, 751–761. 31929948 10.1021/acscatal.9b04221 PMC6945685

[15] X. Xiao , K. Chen , S. F. Xue , Q. J. Zhu , Z. Tao , G. Wei , J. Mol. Struct. 2010, 969, 216–219.

[16] L. L. Liang , X. L. Ni , Y. Zhao , K. Chen , X. Xiao , Y. Q. Zhang , C. Redshaw , Q. J. Zhu , S. F. Xue , Z. Tao , Inorg. Chem. 2013, 52, 1909–1915. 23360231 10.1021/ic302145j

[17] X. L. Ni , X. Xiao , H. Cong , L. L. Liang , K. Cheng , X. J. Cheng , N. N. Ji , Q. J. Zhu , S. F. Xue , Z. Tao , Chem. Soc. Rev. 2013, 42, 9480–9508. 24048328 10.1039/c3cs60261c

[18] R. H. Gao , Y. Huang , K. Chen , Z. Tao , Coord. Chem. Rev. 2021, 437, 213741.

[19] X. Song , M. Cao , R. Chen , H. Wang , H. Li , R. Cao , Chem. Commun. 2021, 57, 2491–2494. 10.1039/d0cc08353d 33538286

[20] Y. Zhang , X. Y. Zhang , K. Chen , W. Y. Sun , ChemSusChem 2021, 14, 1847–1852. 33733591 10.1002/cssc.202100431

[21] A. Day , A. P. Arnold , R. J. Blanch , B. Snushall , J. Org. Chem. 2001, 66, 8094–8100. 11722210 10.1021/jo015897c

[22] J. Kim , I. S. Jung , S. Y. Kim , E. Lee , J. K. Kang , S. Sakamoto , K. Yamaguchi , K. Kim , J. Am. Chem. Soc. 2000, 122, 540–541.

[23] W. S. Jeon , K. Moon , S. H. Park , H. Chun , Y. H. Ko , J. Y. Lee , E. S. Lee , S. Samal , N. Selvapalam , M. V. Rekharsky , V. Sindelar , D. Sobransingh , Y. Inoue , A. E. Kaifer , K. Kim , J. Am. Chem. Soc. 2005, 127, 12984–12989. 16159293 10.1021/ja052912c

[24] J. Lü , J. X. Lin , M. N. Cao , R. Cao , Coord. Chem. Rev. 2013, 257, 1334–1356.

[25] T. Ouyang , H.-J. Wang , H.-H. Huang , J.-W. Wang , S. Guo , W.-J. Liu , D.-C. Zhong , T.-B. Lu , Angew. Chem. Int. Ed. 2018, 130, 16718–16723.

[26] S. L. F. Chan , T. L. Lam , C. Yang , S. C. Yan , N. M. Cheng , Chem. Commun. 2015, 51, 7799–7801. 10.1039/c5cc00566c 25783610

[27] T. Ouyang , C. Hou , J. W. Wang , W. J. Liu , D. C. Zhong , Z. F. Ke , T. B. Lu , Inorg. Chem. 2017, 56, 7307–7311. 28613850 10.1021/acs.inorgchem.7b00566

[28] P. G. Alsabeh , A. Rosas-Hernández , E. Barsch , H. Junge , R. Ludwig , M. Beller , Catal. Sci. Technol. 2016, 6, 3623–3630. 10.1039/c6cc01671e 27301351

[29] Z. Guo , S. Cheng , C. Cometto , E. Anxolabéhère-Mallart , S. M. Ng , C. C. Ko , G. Liu , L. Chen , M. Robert , T. C. Lau , J. Am. Chem. Soc. 2016, 138, 9413–9416. 27443679 10.1021/jacs.6b06002

[30] R. Ziessel , J. Hawecker , J.-M. Lehn , Helv. Chim. Acta 1986, 69, 1065–1084.

[31] Z. Guo , F. Yu , Y. Yang , C. F. Leung , S. M. Ng , C. C. Ko , C. Cometto , T. C. Lau , M. Robert , ChemSusChem 2017, 10, 4009–4013. 28840967 10.1002/cssc.201701354

[32] D. Zhou , X. Li , Q. Zhou , H. Zhu , Nat. Commun. 2020, 11, 2944. 32522995 10.1038/s41467-020-16833-1 PMC7287091

[33] A. McNally , B. Haffemayer , B. S. L. Collins , M. J. Gaunt , Nature 2014, 510, 129–133. 24870240 10.1038/nature13389

[34] M. Nandi , P. Roy , H. Uyama , A. Bhaumik , Dalton Trans. 2011, 40, 12510–12518. 21989952 10.1039/c1dt10157a

[35] P. Xie , J. Ding , Z. Yao , T. Pu , P. Zhang , Z. Huang , C. Wang , J. Zhang , N. Zecher-Freeman , H. Zong , D. Yuan , S. Deng , R. Shahbazian-Yassar , C. Wang , Nat. Commun. 2022, 13, 1375. 35296655 10.1038/s41467-022-28987-1 PMC8927601

[36] T. Jia , D. Meng , R. Duan , H. Ji , H. Sheng , C. Chen , J. Li , W. Song , J. Zhao , Angew. Chem. Int. Ed. 2023, 135, e202216511. 10.1002/anie.202216511 36625466

[37] H. Huang , Y. Zhao , Y. Bai , F. Li , Y. Zhang , Y. Chen , Adv. Sci. 2020, 7, 2000012. 10.1002/advs.202000012 PMC7201256 32382489

[38] P. Liu , X. Zou , X. Y. Meng , C. Peng , X. Li , Y. Wang , F. Zhao , Y. X. Pan , AIChE J. 2023, 69, e18016.

[39] Z. Li , L. Li , D. Hu , C. Gao , J. Xiong , H. Jiang , W. Li , J. Colloid Interface Sci. 2019, 539, 400–413. 30597286 10.1016/j.jcis.2018.12.078

[40] H. You , D. Wu , Z. N. Chen , F. Sun , H. Zhang , Z. Chen , M. Cao , W. Zhuang , R. Cao , ACS Energy Lett. 2019, 4, 1301–1307.

[41] N. J. Wheate , D. P. Buck , A. I. Day , J. G. Collins , Dalton Trans. 2006, 451–458. 16395444 10.1039/b513197a

[42] J. A. Marsella , Kirk-Othmer Encyclopedia of Chemical Technology 2000, 1–9.

[43] L. Q. Chai , H. S. Zhang , J. J. Huang , Y. L. Zhang , Spectrochim. Acta A Mol. Biomol. Spectrosc. 2015, 137, 661–669. 25247838 10.1016/j.saa.2014.08.084

[44] K. R. Grünwald , M. Volpe , P. Cias , G. Gescheidt , N. C. Mösch-Zanetti , Inorg. Chem. 2011, 50, 7478–7488. 21761832 10.1021/ic200279g

[45] L. Troian-Gautier , C. Moucheron , Molecules 2014, 19, 5028–5087. 24759069 10.3390/molecules19045028 PMC6270827

[46] D. Paul Rillema , G. Allen , T. J. Meyer , D. Conrad , Inorg. Chem. 1983, 22, 1617–1622.

[47] I. E. Soshnikov , N. v. Semikolenova , K. P. Bryliakov , A. A. Antonov , W. H. Sun , E. P. Talsi , J. Organomet. Chem. 2019, 880, 267–271.

[48] Y. Yao , Y. Gao , L. Ye , H. Chen , L. Sun , J. Energy Chem. 2018, 27, 502–506.

[49] T. P. Yoon , M. A. Ischay , J. Du , Nat. Chem. 2010, 2, 527–532. 20571569 10.1038/nchem.687

[50] M. Mohan , T. Suzuki , A. K. Nair , S. Pillai , K. G. K. Warrier , U. S. Hareesh , B. N. Nair , J. D. Gale , Phys. Chem. Chem. Phys. 2017, 19, 25564–25573. 28902206 10.1039/c7cp03866f

[51] W. Liu , X. Lu , W. Xue , S. K. Samanta , P. Y. Zavalij , Z. Meng , L. Isaacs , Chem. Eur. J. 2018, 24, 14101–14110. 30044903 10.1002/chem.201802981

[52] J. W. Lee , S. Samal , N. Selvapalam , H. J. Kim , K. Kim , Acc. Chem. Res. 2003, 36, 621–630. 12924959 10.1021/ar020254k

[53] Y. Wang , Y. Liu , L. Wang , S. Perumal , H. Wang , H. Ko , C. L. Dong , P. Zhang , S. Wang , T. T. T. Nga , Y. D. Kim , Y. Ji , S. Zhao , J. H. Kim , D. Y. Yee , Y. Hwang , J. Zhang , M. G. Kim , H. Lee , Nat. Commun. 2024, 15, 6047. 39025876 10.1038/s41467-024-49927-1 PMC11258228

[54] F. F. Chen , L. Zhou , C. Peng , D. Zhang , L. Li , D. Xue , Y. Yu , Appl. Catal. B 2023, 331, 122689.

