# Supplementary material for: Engineering Single Ni Sites on 3D Cage‐like Cucurbit[n]uril Ligands for Efficient and Selective CO2 Photocatalytic Reduction
Source: Angew Chem Int Ed Engl. 2024 Nov 16;64(5):e202417384. doi: 10.1002/anie.202417384 (PMC11773308; doi:10.1002/anie.202417384)
Supplement: Supplementary file 1 — Supporting Information [file ANIE-64-e202417384-s001.pdf]

Angewandte Chemie
Eine Zeitschrift der Gesellschaft Deutscher Chemiker

Supporting Information

Engineering Single Ni Sites on 3D Cage-like Cucurbit[n]uril Ligands for Efficient and Selective CO_2 Photocatalytic Reduction

J. Wang, X. Li, C.-H. Chang, T. Zhang, X. Guan, Q. Liu, L. Zhang, P. Wen, I. Tang, Y. Zhang, X. Yang, J. Tang*, Y. Lan**

Supporting Information

Engineering Single Ni sites on 3D Cage-like Cucurbit[n]uril Ligands for Efficient and Selective CO_2 Photocatalytic Reduction

Jingyi Wang[1, 2, ξ], Xiyi Li[1, 2, ξ, *], Chia-Hao Chang[3], Tianyu Zhang[4], Xuze Guan[5], Qiong Liu[6], Liquan Zhang[3], Ping Wen[7], Ivan Tang[1,2], Yuewen Zhang[7], Xiaofeng Yang[8], Junwang Tang[1, 9 *], Yang Lan[1, 2 *]

[ξ]These authors contributed equally to this paper.

Materials and Methods

Materials

All the reagents and solvents applied in the synthesis and photocatalysis are of analytical grade and purchased from Sigma-Aldrich or Aladdin, which are used as received without further pre-treatment. The ultrapure Milli-Q water (18.2 MΩ·cm^{-1}) is used throughout all experiments.

Synthesis of CB[7]

The synthesis of CB[n] is based on Behrend's synthesis, which involves acid-catalyzed condensation of glycoluril and paraformaldehyde. Before the reaction, glycoluril is formed through the reaction of urea and glyoxal under acidic conditions.[1] After acquiring the CB[n] mixture, CB[n] separation is conducted through multistage recrystallization, which is based on the difference of HCl and H_2O solubility for CB[5], CB[6], CB[7] and CB[8]. CB[5] and CB[7] are water soluble, while CB[6] and CB[8] are barely soluble. Thus, mixture of CB[5] and CB[7] and mixture of CB[6] and CB[8] are primarily separated in the form of filtrate and precipitate respectively by adding ample DI water. The separation of CB[5] and CB[7] is achieved based on their different solubility in ethanol aqueous solution. Ethanol aqueous solution (EtOH: H_2O= 1:1) (ca. 500 mL) is added into the condensed CB[5] and CB[7] aqueous mixture (ca. 200 mL), leading to the separation of CB[5] filtrate and CB[7] precipitate. The separation of CB[6] and CB[8] is following the similar process. (Scheme S1) After vacuum filtration, the purified CB[7] as well as CB[6] and CB[8] are finally acquired and confirmed by ^{1}H NMR using deuterated hydrochloric acid (DCl) as the deuterated solvent. (Figure S1)

Synthesis of CB[7]-Ni

In a typical experiment, CB[7] (148.62 mg) is dispersed in a mixed solvent of ethanol (30 mL) and water (3 mL) via sonication for 10 minutes. The aqueous solution of $Ni(NO_3)_2$ (30 mg/mL; 1.5 mL) with a molar ratio of CB[7] to Ni^{2+} of 1:6 is then added into the dispersion and mixed overnight at room temperature. The solids are washed with ethanol to eliminate excess Ni^{2+} and NO_3^-, followed by centrifuging and vacuum drying at 40 °C, leading to the formation of CB[7]-Ni. The molar ratios of CB[7] and Ni (1:1, 1:2, 1:4, 1:8 and 1:10) in the precursor are also changed and investigated. In the preliminary experiment shown in Figure 1a, CB[7]-M samples (M = Fe, Co, Ni, Cu, Zn) are synthesized following the same procedure, except that the ratio of M and CB[7] is kept at 1:1 in the precursor (Scheme 1).

Synthesis of CB[7]-AMD-Ni

To synthesise the host-guest complex CB[7]-AMD, CB[7] (0.2 mmol, 232.6 mg) is dissolved in 20 mL H_2O, while AMD with the same molar amount (0.2 mmol, 37.5 mg) is dissolved in 2 mL H_2O. The AMD solution is then poured into the CB[7] solution and thoroughly mixed for 1 hour. The mixed solution is then concentrated *in vacuo* to give a white precipitate, which is CB[7]-AMD. This is confirmed by ^{1}H NMR using D_2O as the deuterated solvent (Figure S23). The integration of CB[7]-AMD and Ni^{2+} followed the same

procedure as the combination of CB[7] and Ni^{2+}, namely mixing them thoroughly overnight in a mixed solvent of ethanol (30 mL) and water (3 mL), followed by centrifuging and vacuum drying at 40 °C.

Photocatalytic CO_2 reduction

The photocatalytic experiments are conducted in a septum-sealed quartz reaction bottle with a volume of 160 mL. After the addition of 10 mg catalyst, 15 mg $Ru(bpy)_3Cl_2$ and 12 mL mixed reaction solution, the reactor is purged with CO_2 for 20 minutes while stirring before photocatalytic experiment. The sealed reactor filled with CO_2 is then irradiated using a 300 W Xenon lamp or a 365 nm LED light as the light source. The gaseous products are analysed by gas chromatograph (Shimadzu GC-2010 Pro with a BID-2010 Plus detector).

^{13}C isotope labelling tandem reaction

In a typical experiment, tetramethylpiperidine (TMP) (0.07 mmol, 10 mg) is mixed with $Pd(OAc)_2$ (10 mol%, 1.3 mg) and $Cu(OAc)_2$ (10 mol%, 1.6 mg) in a 10 mL round bottom flask with sealed air condenser. The tandem reaction apparatus is then vacuumed to eliminate air in the system. After photocatalytic $^{12}CO_2$ and $^{13}CO_2$ conversion, the sealed reactor is then respectively connected to the tandem reaction unit by a tube with good airtightness. Then the as-produced ^{12}CO and ^{13}CO as a reactant sufficiently diffused into the tandem reaction apparatus respectively, with the top septum of the apparatus gradually swollen. After swiftly extracting the connecting tube, toluene (0.1 mmol, 1.0 mL) is injected into the sealed unit. Finally, the sealed flask is placed in a pre-heated oil bath at 120 ºC and stirred for 24 hours. After cooling down to room temperature, insoluble compounds are filtered off by passing through a pad of silica gel (silica gel 60 (40-63 μm)) eluting with EtOAc (100%) to trap metal catalysts. The filtrate is collected and concentrated *in vacuo* to give crude compound. This crude product is further investigated by ^{13}C NMR (Figure S6) and high-resolution mass spectrometry (HRMS) (Figure S7). To further confirm the CO product solely from CO_2, after $^{13}CO_2$ isotope labelling experiment the gas product is injected to the GC-MS (Agilent 8890-5977) with proper separation configuration for analysis. A propoak Q column and a molsieve 5A column are used with a backflush system to separate the CO from CO_2.

Materials characterization

The atomic resolution microscopy analysis is performed on a Thermo Fisher Scientific USA Titan Themis Z thermal-field emission microscope with a probe spherical aberration (Cs) corrector working at 300 kV. The X-ray absorption fine structure spectra (Ni K-edge) are collected at 4B9A beamline in Beijing Synchrotron Radiation Facility (BSRF). The storage rings of BSRF are operated at 2.5 GeV with a stable current of 400 mA. The radiation is monochromatized using a Si (111) double-crystal monochromator. The data collection is carried out in fluorescence mode using Lytle detector. All spectra are collected in ambient conditions. The attenuated total reflection Fourier transform infrared spectroscopy (ATR-FTIR) is measured via the Bruker OPUS software at the wavenumber ranging from 400 to 4000 cm^{-1}. In situ ATR-FTIR spectra of CB[7]-Ni at different reaction times under irradiation are measured using a Thermo Scientific Nicolet iS50

FT-IR, equipped with a liquid nitrogen-cooled mercury–cadmium-telluride (MCT) detector. UV-vis diffuse reflectance spectra (DRS) are collected using an Agilent Carry 4000 UV-Vis-NIR spectrophotometer with a diffuse reflectance unit. X-ray photoelectron spectroscopy (XPS) measurement is conducted using the X-ray source of Al k alpha with the energy of 1486.6 eV on Thermo scientific MultiLab 2000, and the results are analysed via Casa XPS software based on the calibration of C (carbon) 1 s peak (binding energy = 284.8 eV). The powder X-ray diffraction (XRD) is measured in a Stoe STADI-P instrument (10° to 40°, step 0.5 ° at 5.0 s/step) using Mo Kα1 (wavelength 0.70930 Å, 50 kV and 30 mA). Photoluminecscence (PL) emission spectra are collected by Renishaw InVia Raman with a 325 nm excitation laser. Electron paramagnetic resonance (EPR) is measured by a JEOL JES-FA200 ESR spectrometer at the temperature of 77 K. Temperature-programmed CO_2 desorption (TPD-CO_2) experiments are performed on a Vodo VDSorb-91i-VAP-HB Chemisorption analyzer. Temperature-programmed H_2O desorption (TPD-H_2O) data are obtained indirectly via using the Pyris 1 TGA-MS (Perkin Elmer & Hiden). CO_2 adsorption isotherms are measured using QuantaChrome Autosorb iQ2 automated gas sorption analyser at 273 K. The actual content of Ni is measured by a Varian 720 Inductively coupled plasma optical emission spectroscopy (ICP-OES) (axial configuration) equipped with the autosampler. Cyclic voltammetry (CV) tests are conducted using an electrochemical workstation (Gamry reference 600 potentiostat) with a standard three-electrode system. The photocatalytic experiments are performed under the irradiation of a 300 W Xe lamp (Beijing Perfectlight, China) or a 365 nm LED light (Beijing Perfectlight). All the gas products are detected by gas chromatography (GC) (Shimadzu GC-2010 Pro with a BID-2010 Plus detector). ^{13}C NMR spectra are obtained on a Bruker Avance 700 spectrometer with TCI cryoprobe at 700 MHz. All the other liquid products are tested by Bruker Avance 400 MHz NMR spectrometer. Liquid chromatography-mass spectrometry (LC-MS) is performed on the LTQ, Agilent 6510 LC Q-TOF system.

DFT simulation

All computations are conducted utilizing Gaussian 09 program package. The optimization of complexes structures and frequency calculation are performed using the B3LYP functional[2,3] and 6-31G* basis set[4] or C, N, O and H, as well as Def2-SVP basis set[5] or Ni and Ru. Long range effects are addressed using Grimme's D3 dispersion correction to describe van der Waals (vdW) dispersion interactions.[6,7] The properties of the excited electronic states and the energies of HOMO/LUMO are calculated via time-dependent DFT (TD-DFT).[8] The influence of the solvent is assessed by solvation model based on density (SMD).[9] The Gibbs free energy change is obtained based on the following equation:

$$\Delta G = \Delta E + \Delta ZPE - T\Delta S$$

Where ΔE is the electronic energy change, ΔZPE is the change of zero point energy, ΔS is the change in entropy. The zero point energy and entropy are obtained by frequency calculation.

Quantum yield (Φ) calculation

Since the incident light cannot be fully absorbed by $[Ru(bpy)_3]Cl_2$, to obtain a more accurate quantum efficiency in the system using $[Ru(bpy)_3]Cl_2$ as photosensitizer, the absorbed photons are estimated following the reported method.[10,11] During the measurement of quantum efficiency, the incident light (λ = 450 nm) intensity is 11.68 mW/cm^2. The light intensity is determined to be 10.02 mW/cm^2 after passing through the reactor filled with CB[7]-Ni mixed solution (CH_3CN:TEOA:H_2O = 4:1:1) in the absence of $[Ru(bpy)_3]Cl_2$. This indicates that the quartz reactor, gas and reaction solution can contribute to some reflection loss or absorption loss. After the addition of $[Ru(bpy)_3]Cl_2$ in the solution, the light intensity after passing through the reaction system is measured as 0.07 mW/cm^2. Therefore, the absorbed light intensity of $[Ru(bpy)_3]Cl_2$ is calculated to be 9.95 mW/cm^2 after subtracting the reflection loss and absorption loss of the quartz, solvent and gas.

Thus, quantum yield (Φ) of the reaction system can be calculated by the following equation:

$$\Phi = \frac{\text{Number of reacted electrons}}{\text{Number of absorbed photons}} \times 100\% = \frac{n_{CO} \times 2 \times N_A}{\Delta I_a \times A \times \frac{\lambda}{hc} \times t} \times 100\%$$

Where n_{CO} is the molar amount of CO yield (13.71 μmol) in one hour; N_A is the Avogadro's number (6.02 × 10^{23}); ΔI_a is the average absorbed light intensity of $[Ru(bpy)_3]Cl_2$ (9.95 mW/cm^2); A is the irradiation area (15.2 cm^2); *h* is the Planck's constant (6.63 × 10^{-34} J·s); *c* is the speed of light (3 × 10^8 m/s); λ is the wavelength of the incident light (λ = 450 nm); t is the time (3600 s). A quantum yield is therefore calculated to be approximately 1.34%.

Cyclic voltammetry (CV)

To prepare the catalyst ink, 7.0 mg of the as-synthesized CB[7]-Ni is thoroughly dispersed via sonication in the mixed solvent of 50 μL of Nafion 117 solution, 200 μL of ethanol and 100 μL of H_2O. The resulting homogeneous white suspension is then uniformly deposited dropwise onto a 1 cm × 1 cm hydrophobic carbon paper and dried using an infrared heat lamp. For the CV measurements, a standard three-electrode system is established, consisting of the catalyst coated carbon paper as the working electrode, non-aqueous Ag/Ag^+ as the reference electrode, and a Pt foil as the counter electrode. The potential for Ag/Ag^+ nonaqueous reference electrode is 0.54 V vs NHE.[12] All the electrodes are inserted into a sealed glass cell containing 30 mL of 0.1 M NBu_4PF_6/anhydrous DMF as the electrolyte. Prior to the CV test, the cell is purged with either Ar or CO_2 gas for 20 minutes. The scan rate for the entire test is set at 100 mV/s.

In-situ EPR experiment

Before the test, 5 mg of CB[7]-Ni with 2 mg of $Ru(bpy)_3Cl_2$ is fully dispersed in CH_3CN. The mixed suspension is injected into the capillary tube, and then either Ar or CO_2 is purged for 3 minutes to remove air inside the tube, respectively. The sealed tube is then inserted into the spectrometer for the measurement at 77 K (liquid nitrogen). The tests are performed under dark condition and light irradiation of Xenon lamp.

In-situ PL experiment

In-situ steady-state PL is measured at the region of 450-750 nm after excitation by a 325 nm laser. A solution (10 mL CH_3CN and 2 mL H_2O) containing 2 mg of $Ru(bpy)_3Cl_2$ is introduced into the cell. The cell is sealed and purged with Ar to eliminate the influence of oxygen from the air. Another solution with additional 5 mg of CB[7]-Ni is used for comparison. After both tests, the CB[7]-Ni-containing system is purged with CO_2 for PL spectrum collection.

Temperature-programmed desorption (TPD)

Prior to TPD-CO_2 test, CB[7]-Ni, CB[7] and $Ni(NO_3)_2$ (ca. 10 mg) are loaded in a U-shaped cylindrical glass microreactor respectively, leading to a fixed clay-bed with a height of 0.1-0.2 cm. The sample is then pre-treated under a He gas flow (50 mL/min) for 2 hours at 250 °C, to remove surface impurities and moisture present on the sample. The clay sample is subsequently cooled down to 40°C under the same He flow. Then, CO_2 gas is purged into the fixed clay-bed system for 60 minutes, while any unabsorbed CO_2 gas is purged away under a He gas flow (50 mL/min) for 2 hours. After reaching the steady state of CO_2 adsorption, the temperature raises gradually to 250 °C at a ramp rate of 20 °C/min under a He gas flow of 50 mL/min, finally leading to the formation of CO_2-TPD curves.

Temperature-programmed H_2O desorption (TPD-H_2O) experiment is conducted using the TGA-MS machine. Before transferring to the TGA-MS machine, the CB[7]-Ni is exposed to the Ar flow with the water steam, which is set at a flow rate of 20.0 ml/min for a duration of 180 minutes. After the adsorption of water, CB[7]-Ni is placed in the sample chamber of the TGA-MS machine. The temperature gradually rises from 25 °C to 200 °C at a ramp rate of 5 °C/min under a 20 mL/min N_2 gas flow. The desorption of water is analyzed by an on-line MS machine.

In-situ ATR-FTIR experiment

Prior to the ATR-FTIR test, the photosensitizer(($Ru(bpy)_3$)Cl_2, 15 mg) and catalyst (10 mg) are thoroughly dispersed in a mixed solvent of CH_3CN and H_2O (12 mL, v/v=5:1). The mixture is then transferred to a quartz reaction cell designed specifically for in situ liquid sample testing. The cell is sealed with a septum, through which a needle is inserted at the top. During the in-situ ATR-FTIR measurements, CO_2 is continuously introduced into the cell through a needle to minimize the interference from air in the CO_2 photoreduction process. A 300 W Xe lamp is used as light source. Background signals are recorded before illumination, followed by the collection of test signals at various time intervals under illumination.

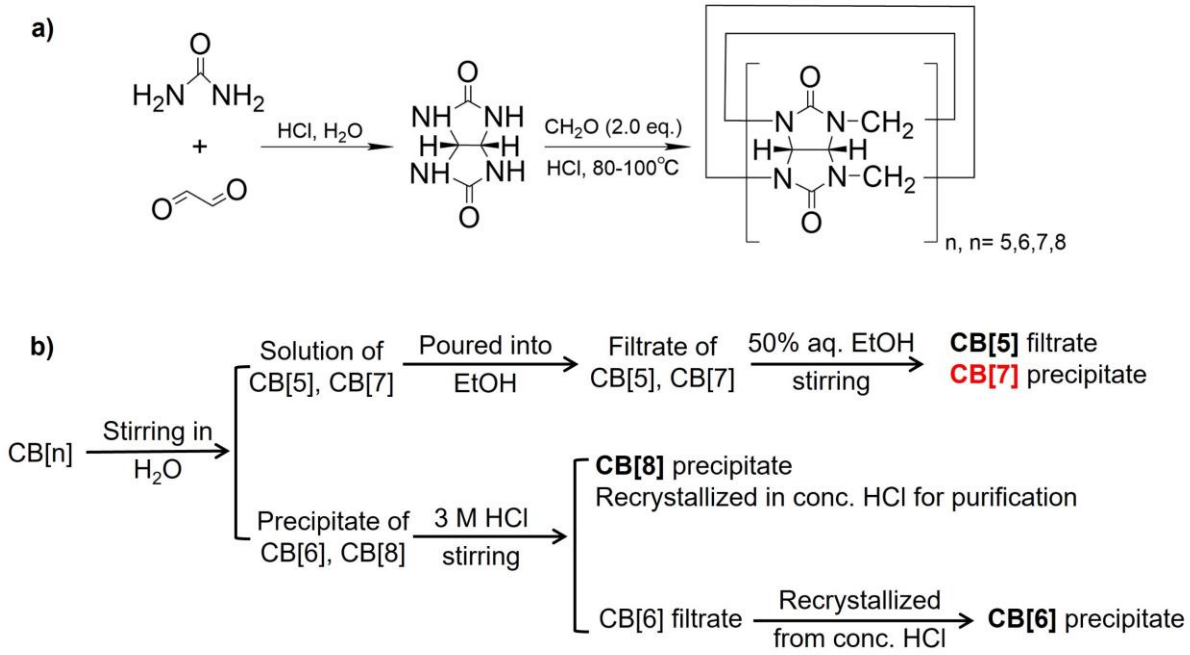

Scheme S1. General procedure for a) synthesis of CB[n]; b) separation of CB[n] to obtain CB[7].

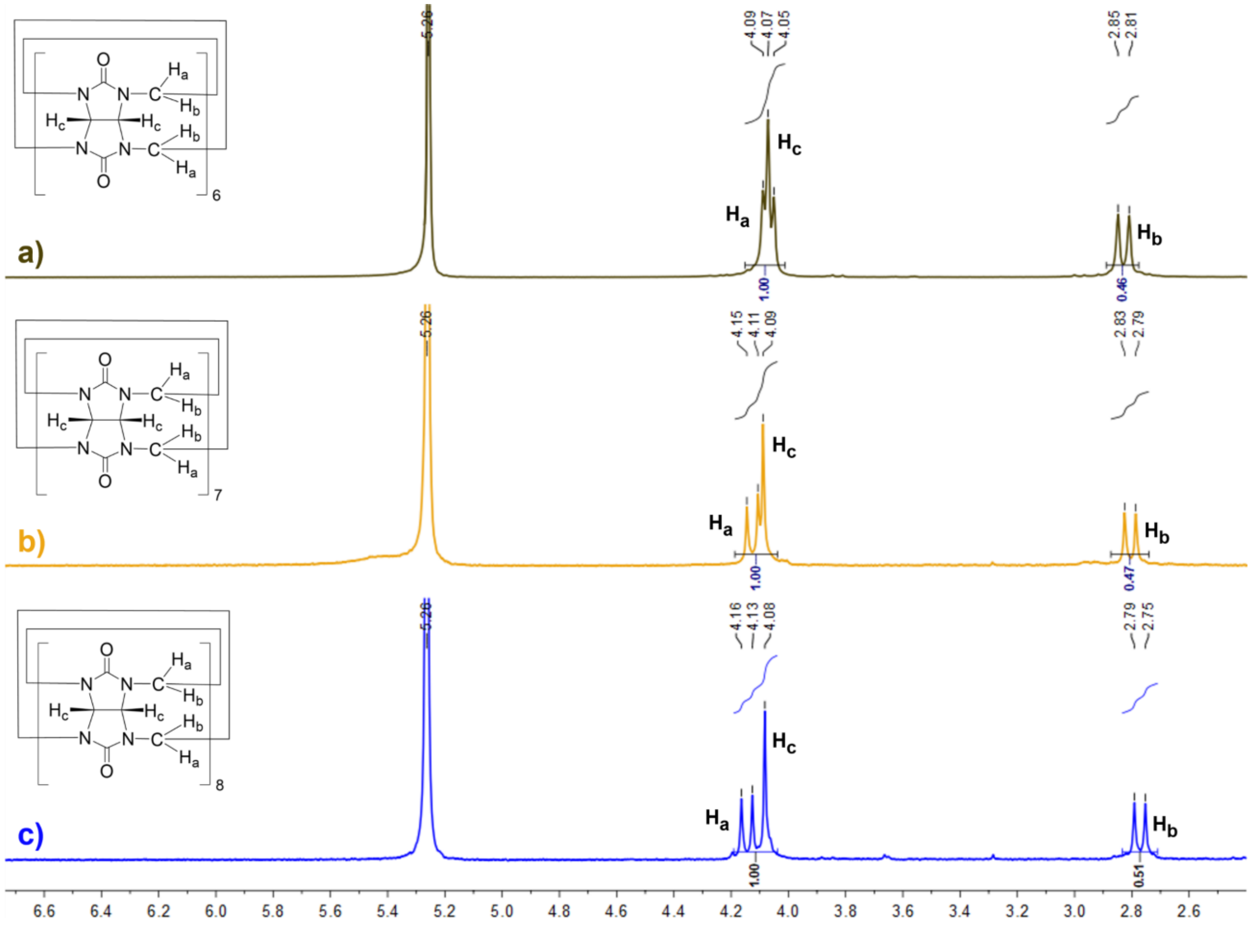

Figure S1. The ^{1}H NMR spectra of purified a) CB[6], b) CB[7] and c) CB[8] using deuterated chloride (DCl) (20 wt% in D_2O) as the solvent.

Due to the insolubility of CB[6] and CB[8] in H_2O, 20% DCl is used as the deuterated solvent to dissolve CB[6], CB[7] and CB[8] for comparison under the same condition. As shown in Figure S1a-c, the typical doublets of CB[n] are all located at ca. 2.8 ppm, without the existence of multi-peaks.[13] The doublets of CB[8], CB[7] and CB[6] in Figure S1c, Figure S1b and Figure S1a, respectively, are also consecutive, with the chemical shift from upfield to downfield. Besides, the integral areas of the consecutive triplets at 4.0-4.2 ppm (H_a + H_c) are twice the integral areas of the doublets of CB[6], CB[7] and CB[8] respectively. These results indicate the effective purification of CB[6], CB[7] and CB[8].[14]

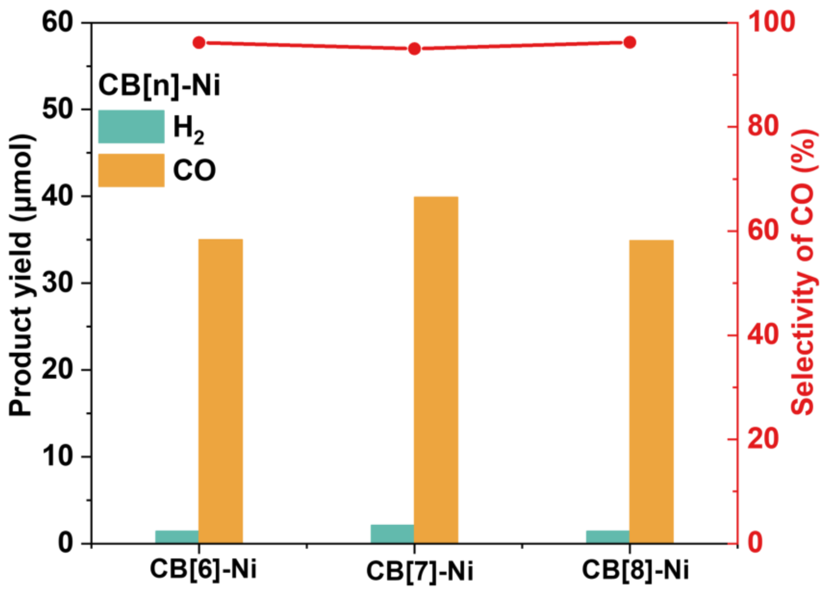

Figure S2. Product yields and CO selectivity of CB[6]-Ni, CB[7]-Ni and CB[8]-Ni in photocatalytic CO_2 reduction. [Reaction condition: 10 mg catalyst, 15 mg $[Ru(bpy)_3]Cl_2$; 12 mL mixed solvent (CH_3CN:TEOA:H_2O = 4:1:1); reaction time 0.5 h; 300 W Xe lamp of full arc spectrum]

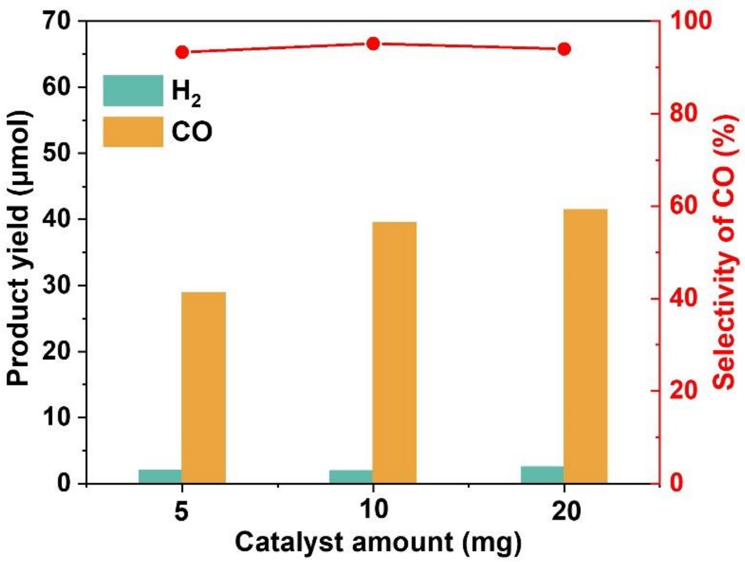

Figure S3. Product yields in photocatalytic CO_2 reduction for CB[7]-Ni with different catalyst amounts [Reaction condition: 15 mg $[Ru(bpy)_3]Cl_2$; 12 mL mixed solvent (CH_3CN:TEOA:H_2O = 4:1:1); reaction time 0.5 h; 300 W Xe lamp (λ > 420 nm)]

Different catalyst masses of CB[7]-Ni were further optimized in photocatalytic CO_2 reduction as shown in Figure S3. When the catalyst amount is increased from 5 mg to 10 mg, the CO yield increased from 28.9 µmol to 39.4 µmol, while the H_2 yield remains relatively unchanged, resulting in an increase in CO selectivity from 93.3% to 95.1%. Further increasing the catalyst amount to 20 mg merely results in a small difference in CO yield (41.4 µmol). Therefore, 10 mg of CB[7]-Ni is chosen for further investigation in photocatalytic CO_2 reduction.

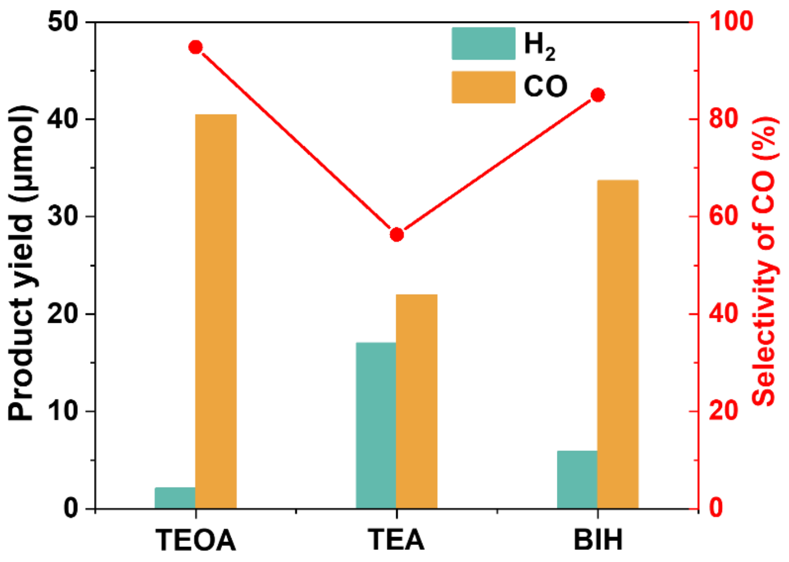

Figure S4. Product yields in photocatalytic CO_2 reduction for CB[7]-Ni using different hole scavengers. [Reaction conditions: 10 mg catalyst, 15 mg $[Ru(bpy)_3]Cl_2$; 12 mL mixed solvents of CH_3CN:TEOA:H_2O (4:1:1) (left), CH_3CN:TEA:H_2O (4:1:1) (middle) and CH_3CN:H_2O (4:1) + 0.1 M BIH (right) respectively; 300 W Xe lamp (λ > 420 nm)]

As shown in Figure S4, using TEOA as the sacrificial agent, CB[7]-Ni achieves a high CO yield of 39.4 μmol· h^{-1} with a remarkable CO selectivity of 95.0% in the repeated experiment under visible irradiation. When TEOA is replaced by using triethylamine (TEA) as the sacrificial agent, CB[7]-Ni results in a comparable H_2 yield (17.1 μmol) and a CO yield (21.9 μmol), with the latter being almost half the amount obtained when using TEOA. In contrast, when 1,3-dimethyl-2-phenyl-2,3-dihydro-1H-benzo[d] imidazole (BIH) is used as the sacrificial reductant, the CO yield slightly decreases to 33.7 μmol, while the H_2 yield increases to 5.8 μmol, resulting in a CO selectivity of 85.0%.

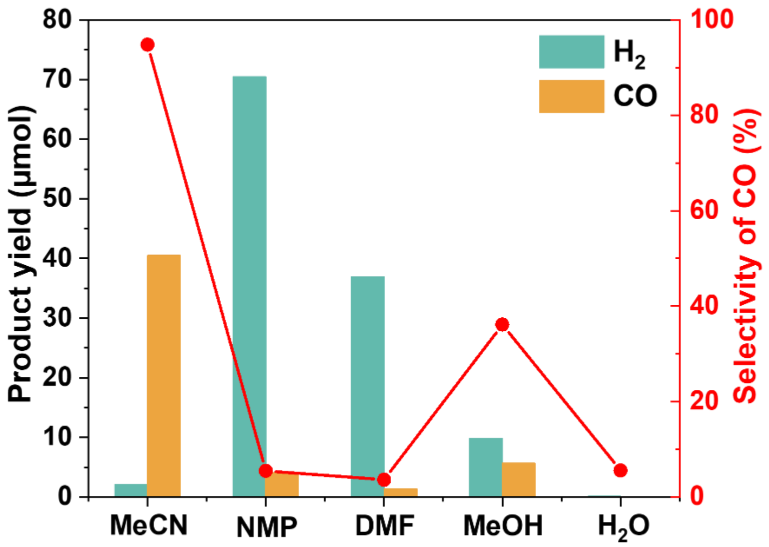

Figure S5. Product yields in photocatalytic CO_2 reduction for CB[7]-Ni using different solvents. [Reaction conditions: 10 mg catalyst, 15 mg $[Ru(bpy)_3]Cl_2$; 12 mL mixed solutions containing different solvents (MeCN/ NMP/ DMF/ MeOH/ H_2O), TEOA and H_2O, respectively (solvent :TEOA :H_2O = 4:1:1); 300 W Xe lamp (λ > 420 nm)]

Different solvents have been investigated as shown in Figure S5. N-methyl-2-pyrrolidone (NMP) is initially chosen as the solvent for comparison due to its similar behavior to that of MeCN.[15,16] When the solvent of MeCN is replaced by NMP, CB[7]-Ni shows a higher H_2 yield (70.5 μmol), whereas only a small amount of CO (4.0 μmol) is observed. When N,N-Dimethylformamide (DMF) is used as the solvent instead of MeCN, the reaction products are similarly dominated by H_2 production (36.8 μmol), with a small amount of CO (1.3 μmol) also formed. When the solvent is changed to methanol (MeOH), low yields of H_2 (9.8 μmol) and CO (5.6 μmol) are observed. In the case of H_2O as the solvent, the yields of both H_2 and CO are extremely low (< 0.1 μmol).

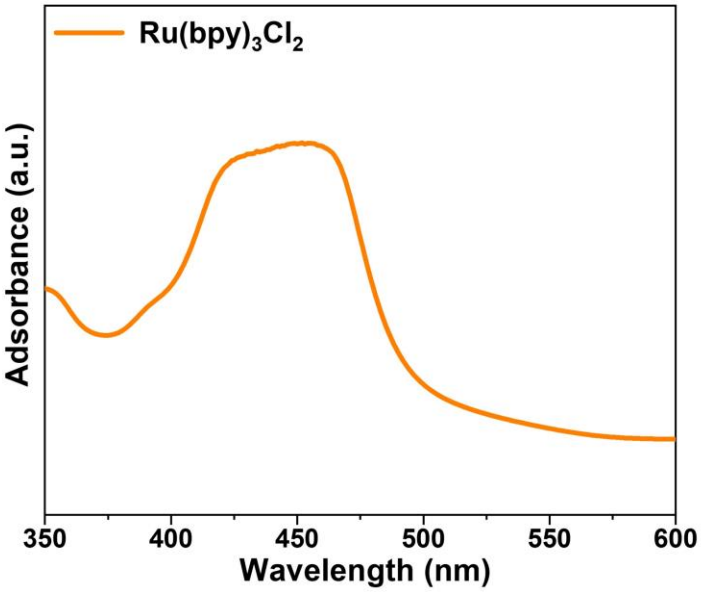

Figure S6. UV-Vis absorption spectrum of $[Ru(bpy)_3]Cl_2$.

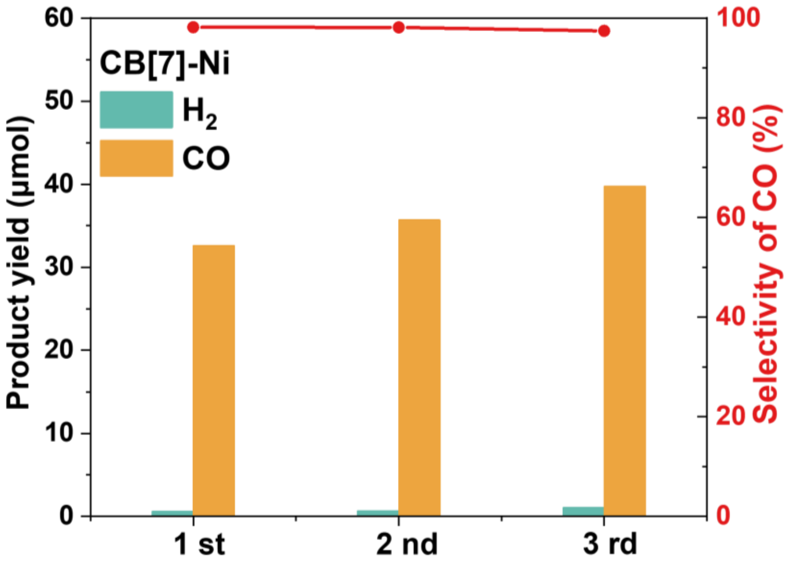

Figure S7. Product yields and CO selectivity of three CB[7]-Ni samples synthesized at different batches in photocatalytic CO_2 reduction. [Reaction condition: 10 mg catalyst, 15 mg $[Ru(bpy)_3]Cl_2$; 12 mL mixed solvent (CH_3CN:TEOA:H_2O = 4:1:1); reaction time 0.5 h; 300 W Xe lamp (λ > 420 nm)]

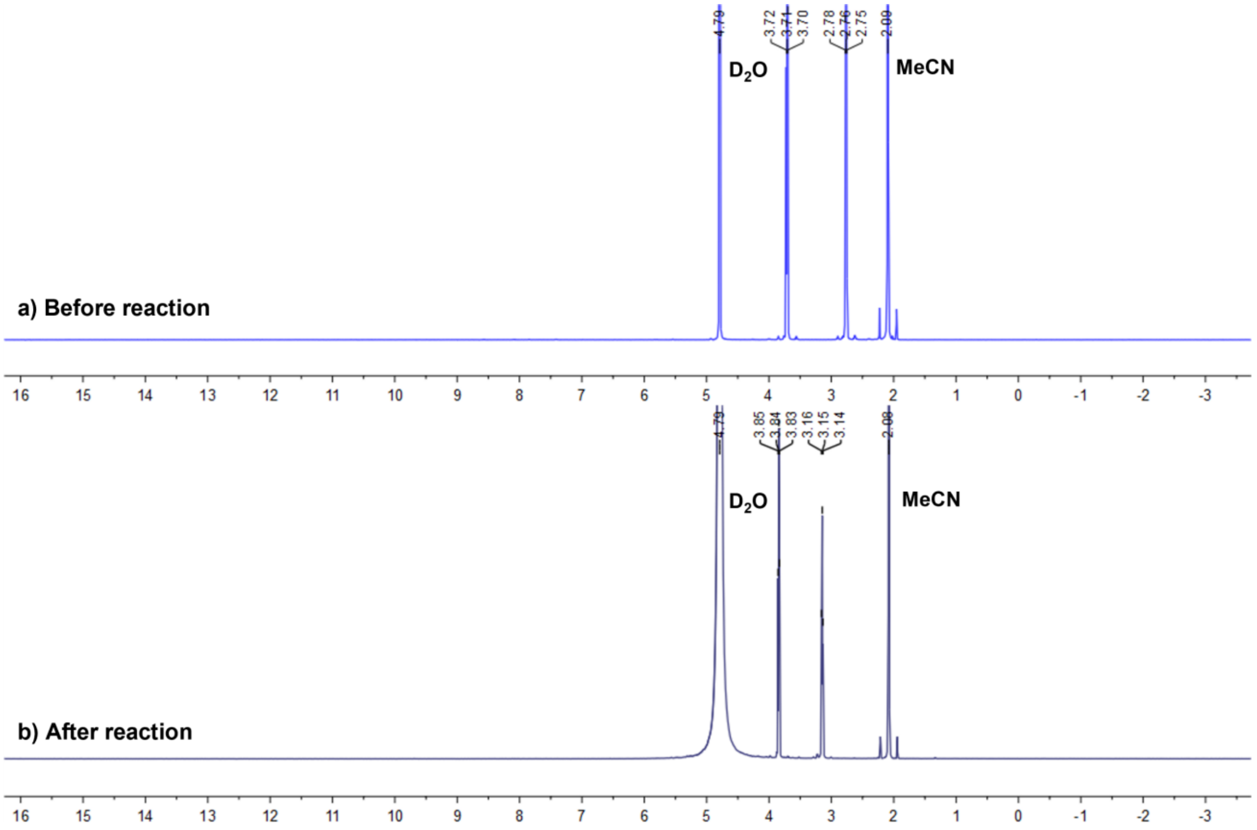

Figure S8. ^{1}H NMR spectra for the liquid mixture of CH_3CN and TEOA containing CB[7]-Ni and $[Ru(bpy)_3]Cl_2$ using D_2O as the deuterated solvent a) before photocatalytic CO_2 reduction; b) after photocatalytic CO_2 reduction.

The characteristic ^{1}H NMR peaks of TEOA, $[Ru(bpy)_3]Cl_2$ and CH_3CN are displayed in Figure S8. After photocatalytic CO_2 reaction, the characteristic peaks of TEOA show the downfield chemical shift due to the oxidation of TEOA. No other products (e.g., HCOOH, CH_3OH etc.) can be observed in liquid phase. The results demonstrate the dependable high selectivity of CO product from CO_2 conversion.

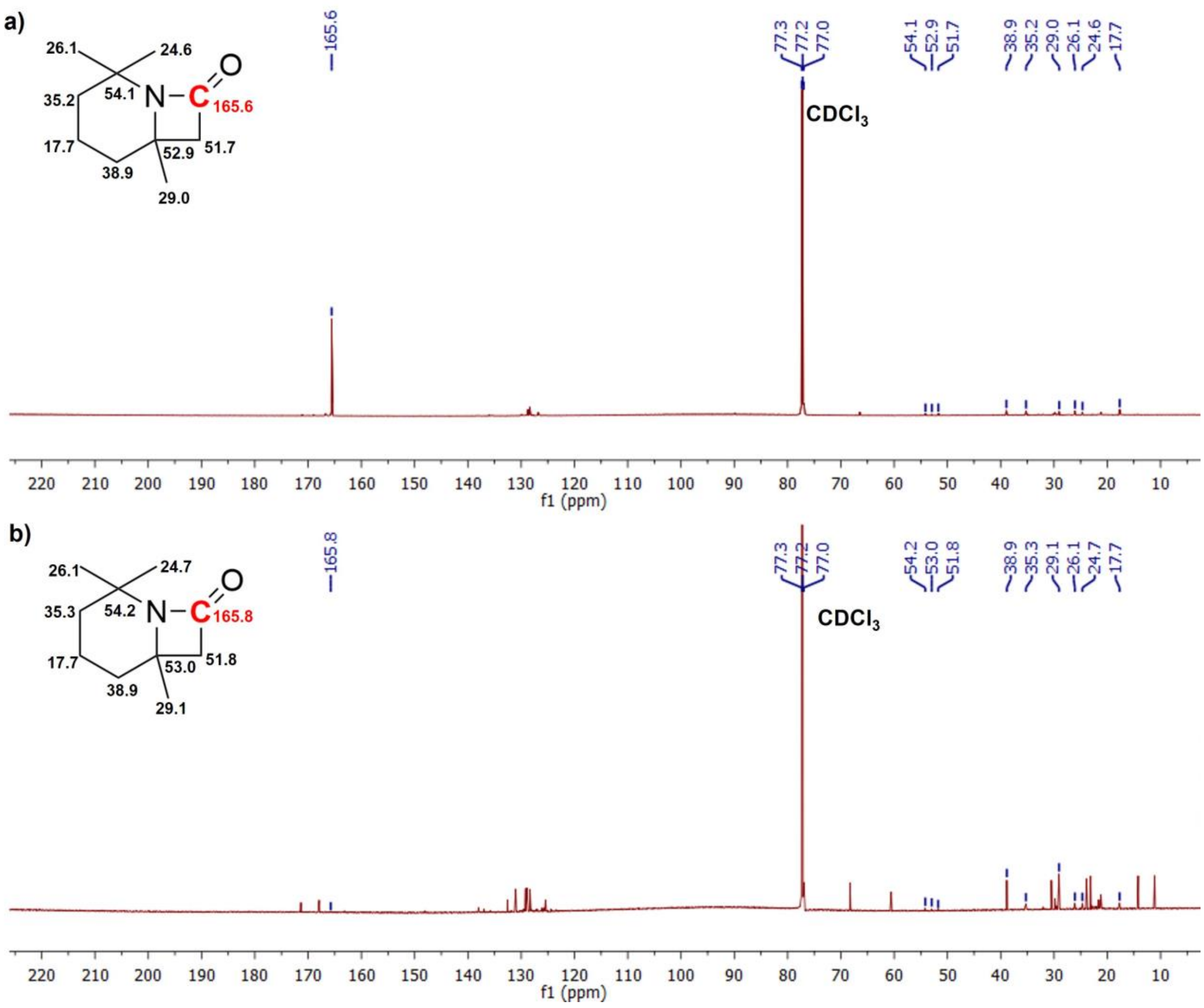

Figure S9. ^{13}C NMR (175 MHz, Chloroform-*d*) spectra of a) ^{13}CO and b) ^{12}CO inserted β-lactam.

For ^{13}CO inserted β-lactam, the carbon of carbonyl is displayed at δ=165.6 ppm, which shows ca. 20 times higher intensity than the carbon of carbonyl displayed at δ=165.8 ppm for ^{12}CO inserted β-lactam. All the peaks agree with the literature.[17]

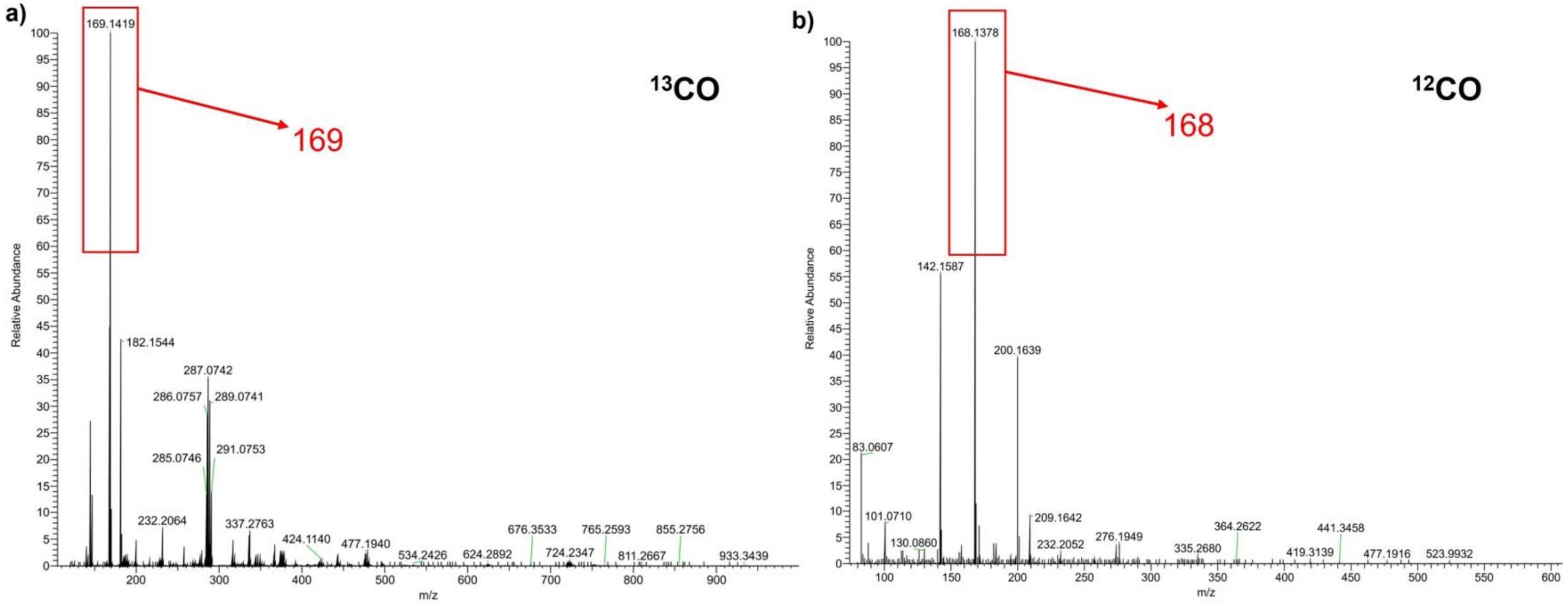

HRMS (ESI+) [M + H]$^+$ Calcd. for $C_{10}H_{18}NO$ = 169.1416, found = 169.1419.

HRMS (ESI+) [M + H]$^+$ Calcd. for $C_{10}H_{18}NO$ = 168.1383, found = 168.1378.

Figure S10. High Resolution Mass Spectrometry (HRMS) (ESI+) [M + H]$^+$ of a) ^{13}CO inserted β-lactam; b) ^{12}CO inserted β-lactam.

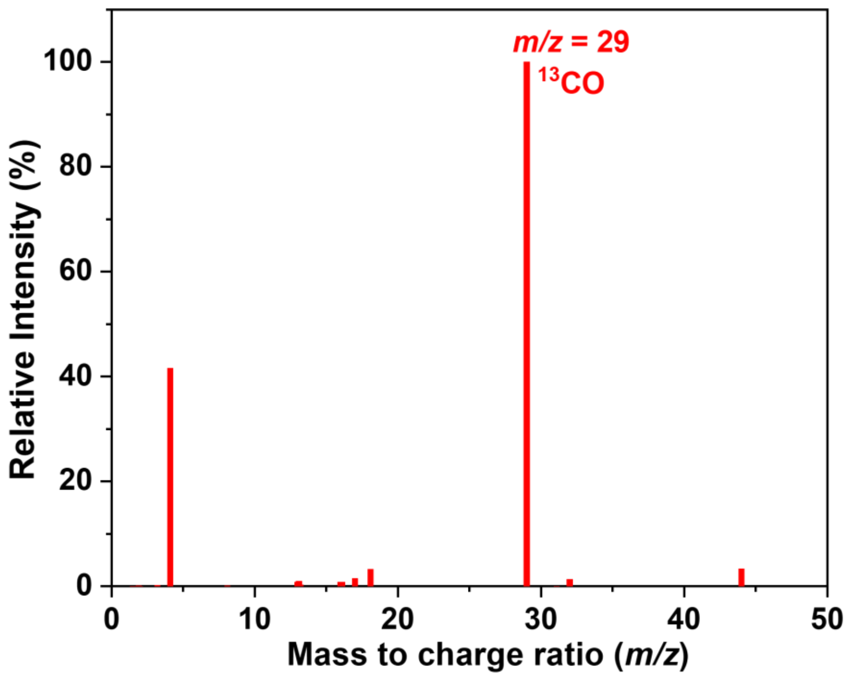

Figure S11. Mass spectrum of ^{13}CO product from photocatalytic $^{13}CO_2$ reduction on CB[7]-Ni.

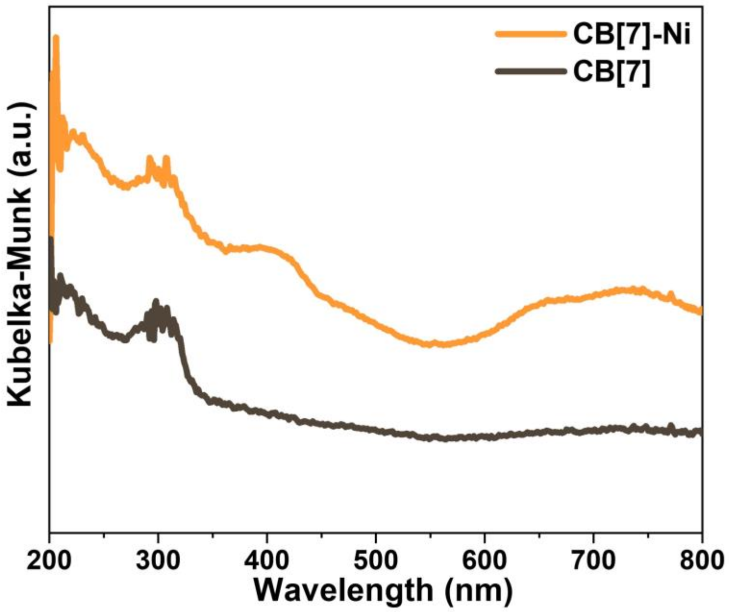

Figure S12. UV-vis diffuse reflectance spectra of CB[7] and CB[7]-Ni.

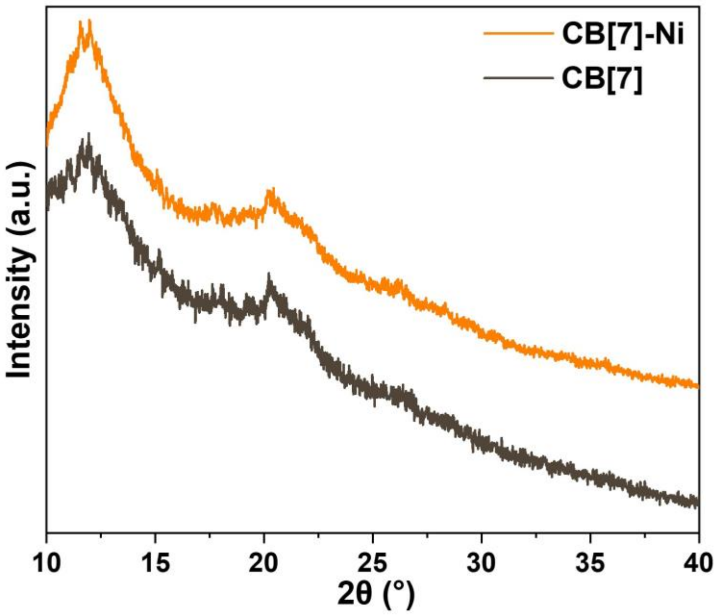

Figure S13. XRD spectra of CB[7] and CB[7]-Ni.

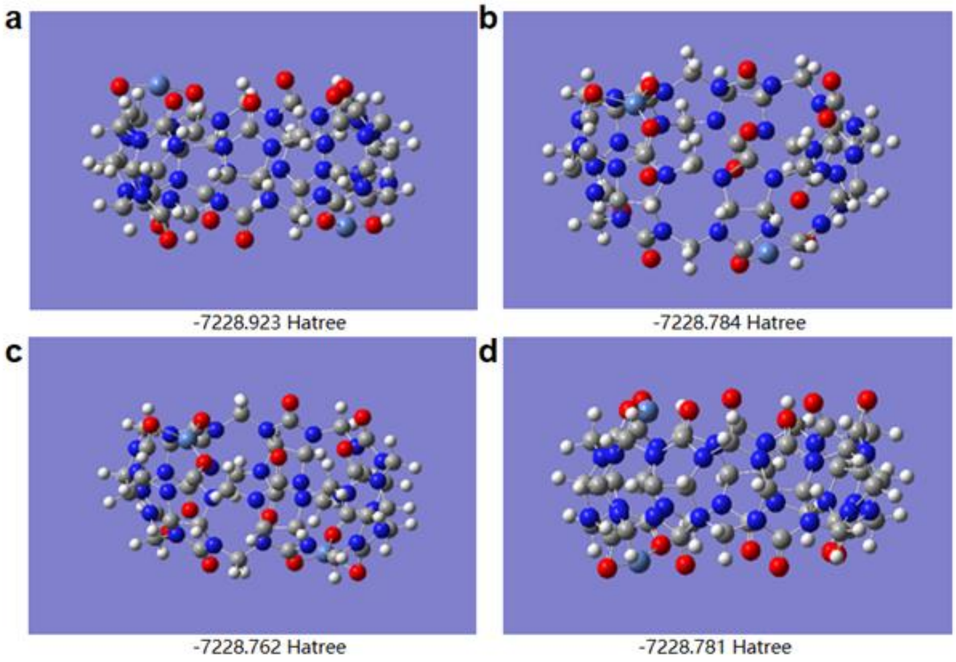

Figure S14. Possible structure of CB7-[Ni] (a) Ni-2O/Ni-2O opposite side, (b) Ni-2O/Ni-3O opposite side (c) Ni-3O/Ni-3O opposite side (d) Ni-2O/Ni-2O same side. The energy is presented below each structure. (Red: oxygen; Navy blue: nitrogen; Dark grey: carbon; White: hydrogen; Light blue: nickel)

During the DFT simulation, the positioning of the two Ni ions at the center of the two portals of the CB[7] molecule lacks stability. Even when this configuration is used as the initial state, the two Ni ions will migrate laterally to coordinate with 2 oxygen atoms following optimization. The side coordination structures are further carefully investigated as shown in Figure S14. Upon structure optimizations, both Ni ions exhibit a tendency to coordinate with two or three oxygen atoms of CB[7]. The structure where both Ni ions coordinate with 2 oxygen atoms (Ni-2O/2O) has the lowest energy. In contrast, the structure where both Ni ions coordinate with 3 oxygen atoms (Ni-3O/3O) has an energy that is higher by 0.161 Hartree. A combination structure where one Ni ion coordinates with 2 oxygen atoms and the other with 3 oxygen atoms (Ni-2O/3O) has an energy that is 0.139 Hartree higher, placing it between the Ni-2O/2O and Ni-3O/3O structures in terms of energy. Interestingly, it is found that the upper Ni and the bottom Ni can be located

either in the same perpendicular direction or at opposite corners along the center symmetric axis, with both structures exhibiting similar energy levels. This is consistent with the dynamic coordination nature between Ni and the carbonyl groups at the portal of CB[7].[18,19] Based on energy minimization principle, it is suggested that two Ni ions coordinate with two oxygen atoms respectively at both portals of CB[7].

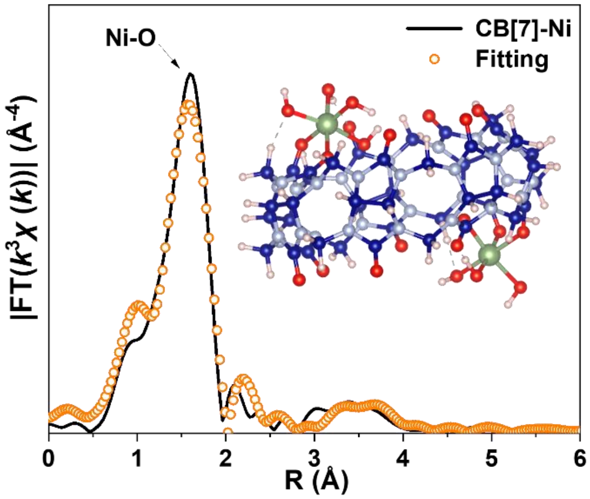

Figure S15. Experimental and EXAFS fitting spectrum of CB[7]-Ni at the R space (inset, DFT-optimized structure of CB[7]-Ni coordination complex) (Red: oxygen; Navy blue: nitrogen; Dark grey: carbon; White: hydrogen; Green: nickel)

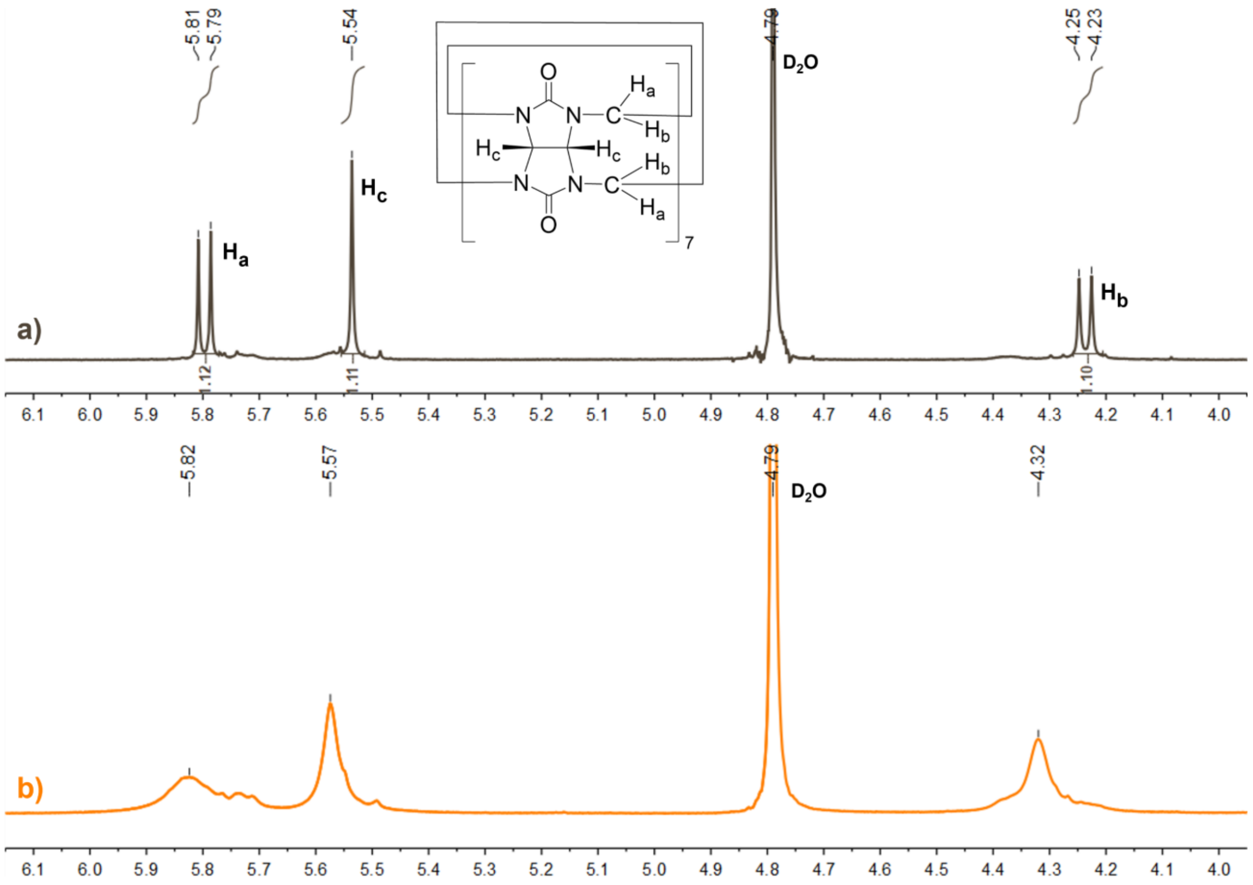

Figure S16. The ^{1}H NMR spectra of a) CB[7] and b) CB[7]-Ni using D_2O as the deuterated solvent.

CB[7] and CB[7]-Ni are dissolved in D_2O under the same condition for the comparison of ^{1}H NMR spectra. In Figure S16a, the integral area of the singlet H_c is almost the same as the integral areas of two respective doublets H_a and H_b from the diastereotopic -CH_2 group. The peak positions are also almost the same as the positions displayed in other literature using D_2O as the deuterated solvent.[20] After the combination of CB[7] and Ni ions, both doublets H_a and H_b merge respectively and exhibit line broadening in Figure S16b, with a notable downfield shift on H_b. This indicates the interactions of CB[7] and Ni ions, and changes of electronic structure in CB[7]-Ni.[21]

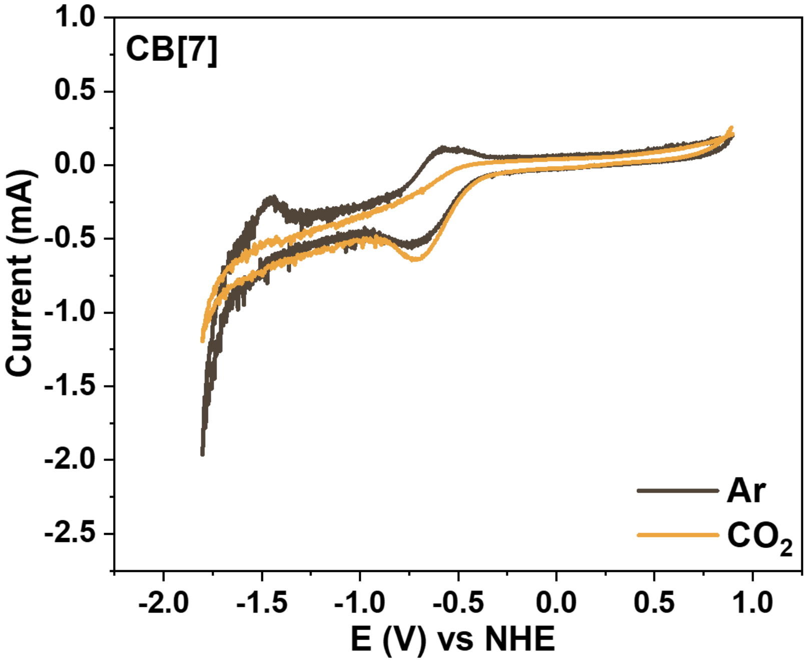

Figure S17. Cyclic voltammogram of CB[7] under Ar atmosphere and CO_2 atmosphere in 0.1 M NBu_4PF_6/DMF solution (pH = 9.9) with scan rate at 100 mV· s^{-1}.

Careful inspection of the solution pH (pH=9.9), this small peak can be attributed to the proton reduction. The calculation process is shown below:[22]

$2H^+ + 2e^- \rightarrow H_2(g)$ $E^0(V) = 0$ V vs. NHE at pH = 0;

$E^0(pH) = E^0(pH = 0) – 0.059$ pH $= 0 – 0.059 \times 9.9 = -0.5841$ V.

This value is highly consistent with the position (-0.59 V) of the peak observed in our CV tests. Therefore, it is reasonable to assign this peak to the proton reduction, which might be from moisture absorption in the hydroscopic DMF solution.[23]

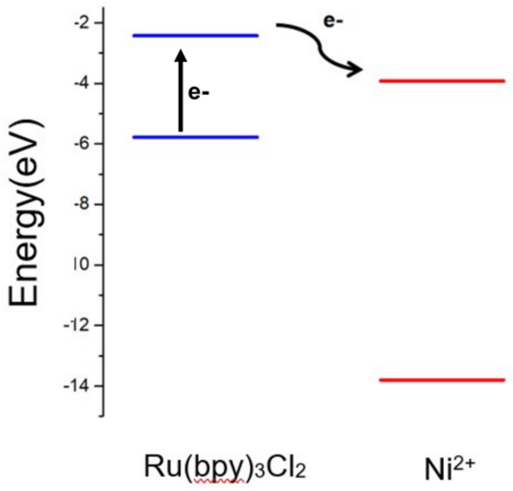

Figure S18. Relevant energy levels of the $Ru(bpy)_3Cl_2$ and Ni^{2+} obtained via DFT simulation.

DFT simulation is used to determine and compare the energy level of the excited states of $Ru(bpy)_3Cl_2$ and the empty orbital of Ni(II) under the same vacuum energy level. The Lowest Unoccupied Molecular Orbital (LUMO) of $Ru(bpy)_3Cl_2$ is as high as -2.43 eV. After light excitation, the electrons can be excited to this energy level, which is higher than the energy level of the unoccupied orbital of Ni(II). Therefore, the photoelectrons transfer from $Ru(bpy)_3Cl_2$ to Ni(II) is thermodynamically feasible.

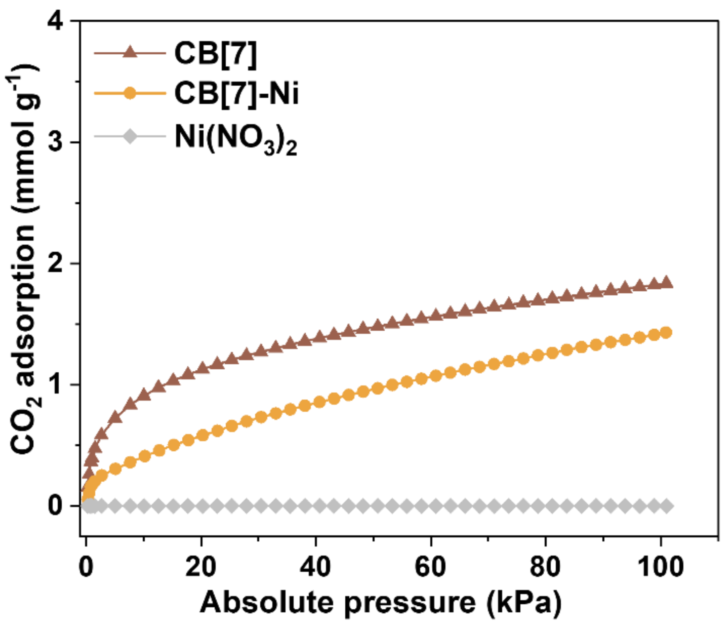

Figure S19. CO_2 adsorption curves of CB[7], CB[7]-Ni and $Ni(NO_3)_2$ at 0 °C.

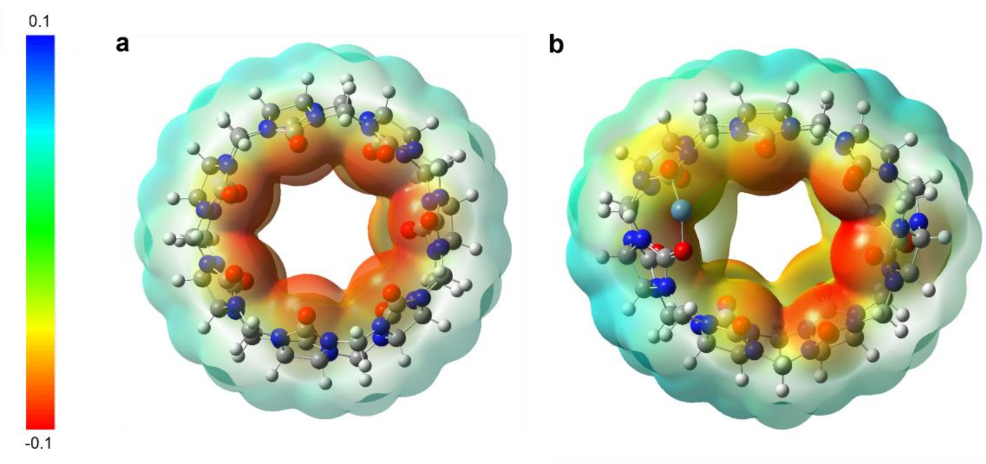

Figure S20. Calculated charge state distribution of (a) CB[7] and (b) CB[7]-Ni (Red: oxygen; Navy blue: nitrogen; Dark grey: carbon; White: hydrogen; Light blue: nickel)

An exhaustive DFT simulation to compare the changes in charge states of CB[7] after coordination with Ni has been provided. As shown in Figure S20, the electrostatic potential maps reveal that anchoring the Ni atom induces a slight deformation of the CB[7] cage and causes a local change in charge properties, with an approximate 10% variation in the electronegativity of oxygen (O) and the electropositivity of hydrogen (H) between CB[7] and CB[7]-Ni. This alteration is relatively minor and does not significantly impact the CO_2 adsorption properties of the CB[7] cage.

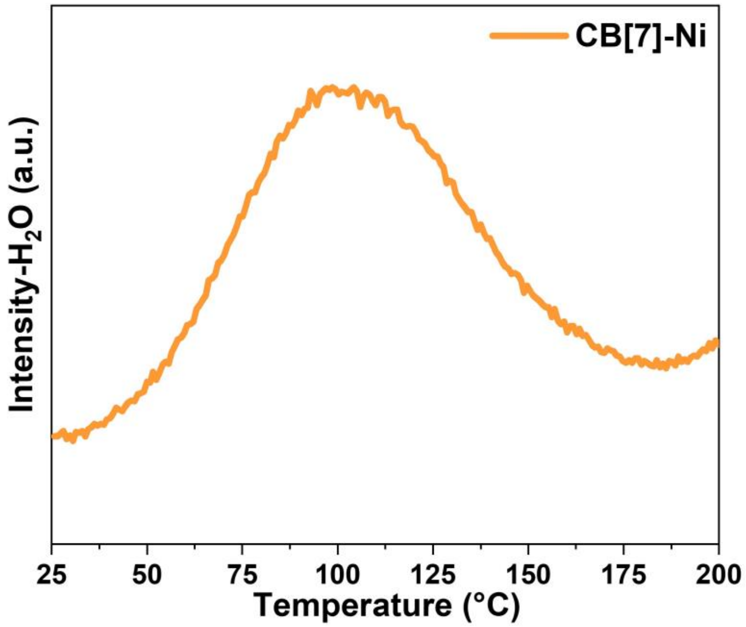

Figure S21. TPD-H_2O profile of CB[7]-Ni.

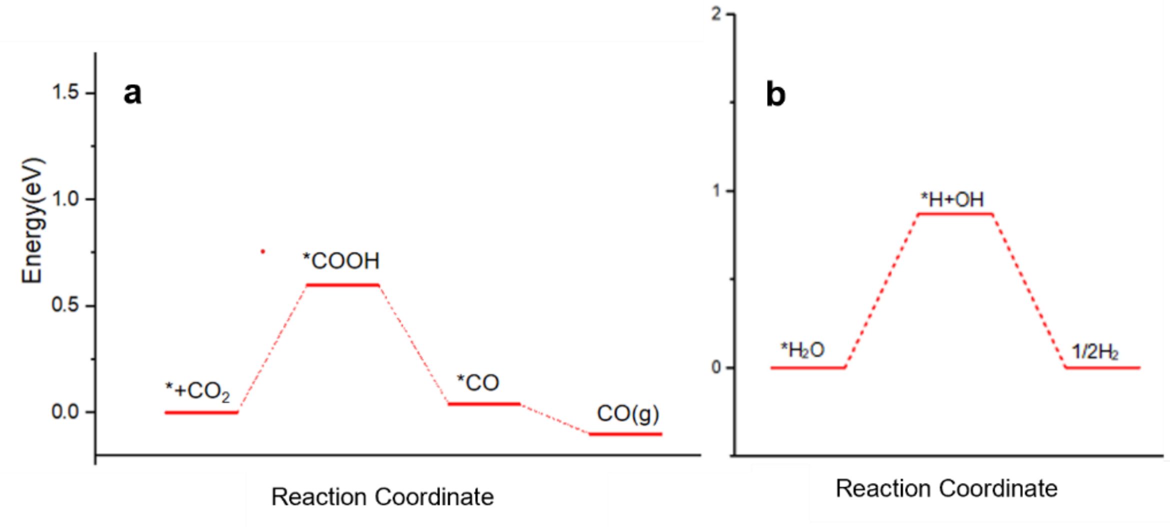

Figure S22. Potential energy diagram of CB[7]-Ni for (a) photocatalytic CO_2-to-CO reduction and (b) hydrogen evolution reaction

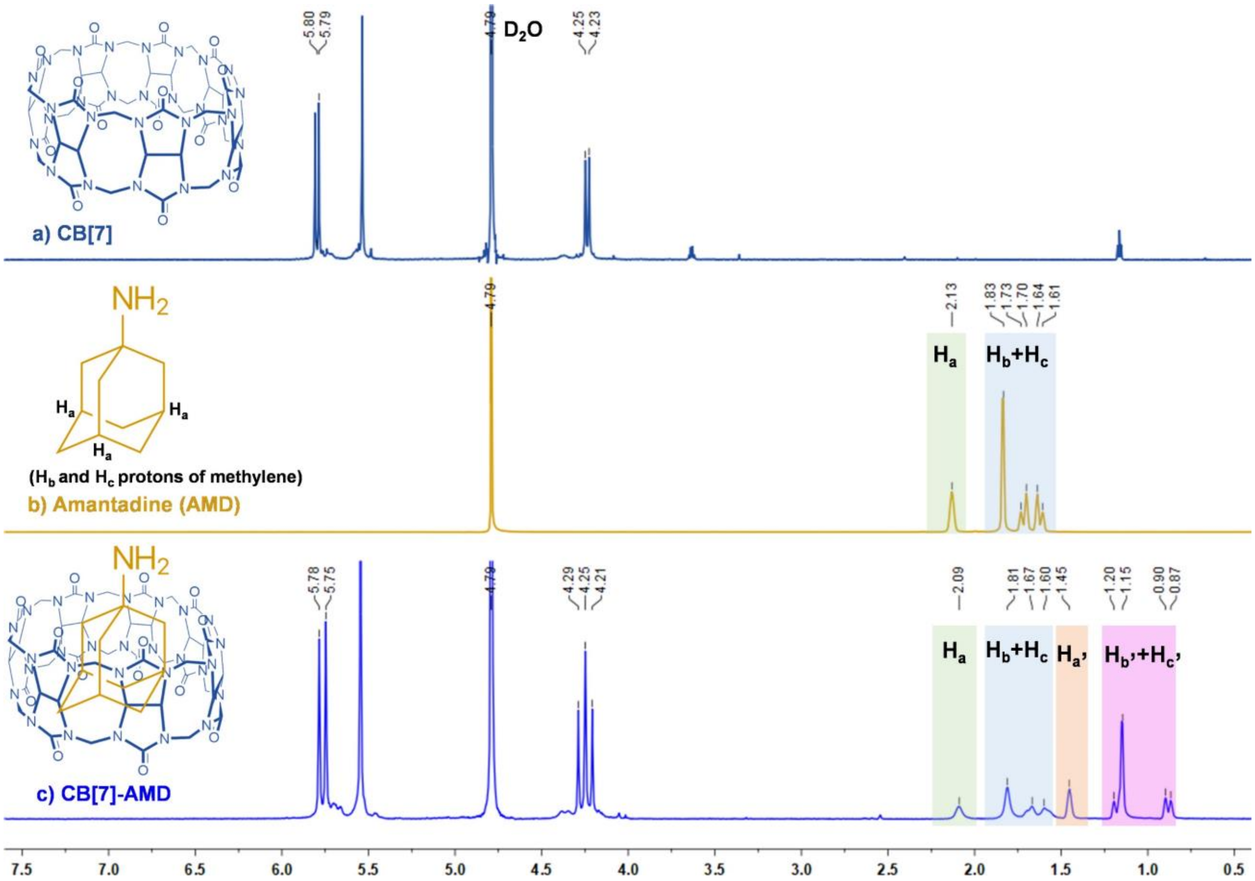

Figure S23. The ^{1}H NMR spectra of a) CB[7], b) AMD and c) CB[7]-AMD using D_2O as the deuterated solvent.

The ^{1}H NMR spectrum of commercial AMD displays the chemical shift in two specific shaded areas, where H_a is assigned as the protons of methine at δ 2.13 (br s, 3H), and H_b+ H_c is assigned as the protons of methylene at δ 1.60-1.90 (m, 12H) in AMD. The protons of $-NH_2$ are not observed in NMR due to active protons. After treatment of CB[7] with AMD, the two shaded areas split into four shaded areas as shown in CB[7]-AMD. The emerged two new areas of H_a’ and H_b’+ H_c’ indicate the host-guest inclusion complexation.[24] The existing shaded areas of H_a and H_b+ H_c represent a small amount of AMD that is not inside the CB[7] cavity. On the other hand, the typical doublet of CB[7] at ca. 4.25 ppm splits into triplet in CB[7]-AMD, with a slight chemical shift. This also indicates the effective interaction between CB[7] and AMD. The result corresponds well with the previous report. [20,24]

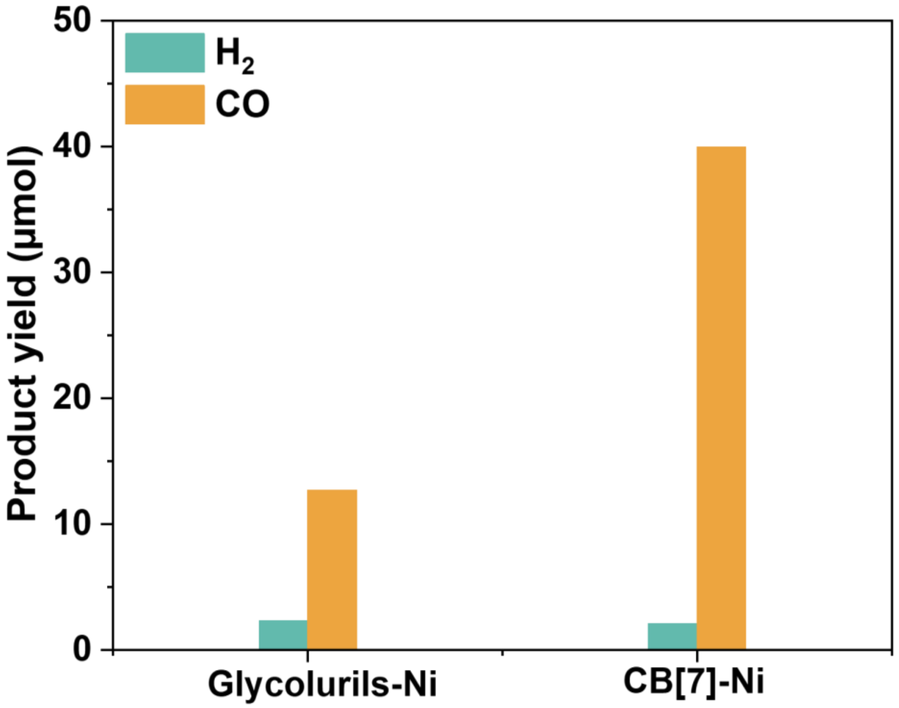

Figure S24. Product yields of glycolurils-Ni and CB[7]-Ni in photocatalytic CO_2 reduction. [Reaction condition: 10 mg catalyst, 15 mg $[Ru(bpy)_3]Cl_2$; 12 mL mixed solvent (CH_3CN:TEOA:H_2O = 4:1:1); reaction time 0.5 h; 300 W Xe lamp (full arc)]

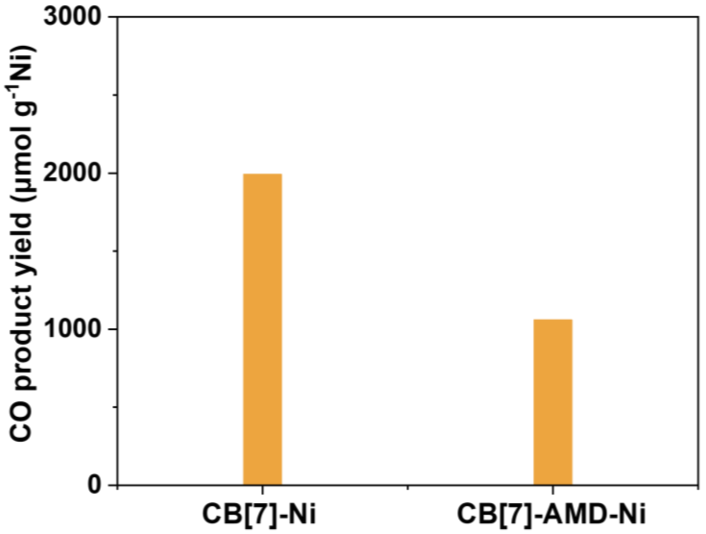

Figure S25. CO product yield of CB[7]-Ni and CB[7]-AMD-Ni in photocatalytic CO_2 reduction. [Reaction condition: 10 mg catalyst, 15 mg $[Ru(bpy)_3]Cl_2$; 12 mL mixed solvent (CH_3CN:TEOA:H_2O = 4:1:1); reaction time 0.5 h; 300 W Xe lamp (λ > 420 nm)]

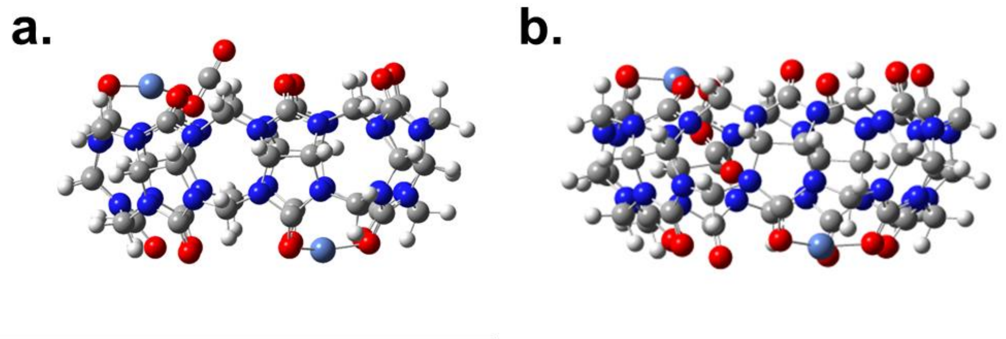

Figure S26. The CO_2 adsorption configuration over CB[7]-Ni: CO_2 outside the cage (left) and CO_2 inside the cage (right) (Red: oxygen; Navy blue: nitrogen; Dark grey: carbon; White: hydrogen; Light blue: nickel)

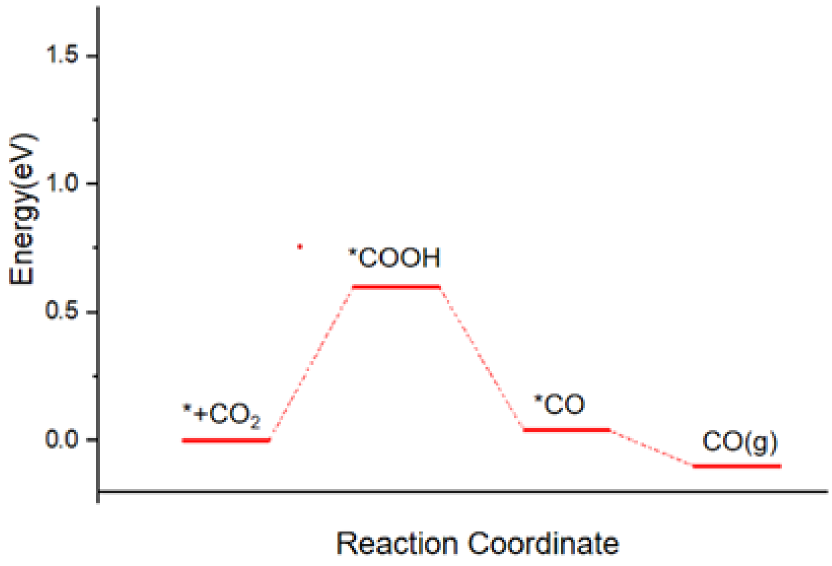

Figure S27. Energy profile of CO_2 to CO reaction pathway within CB[7]-Ni.

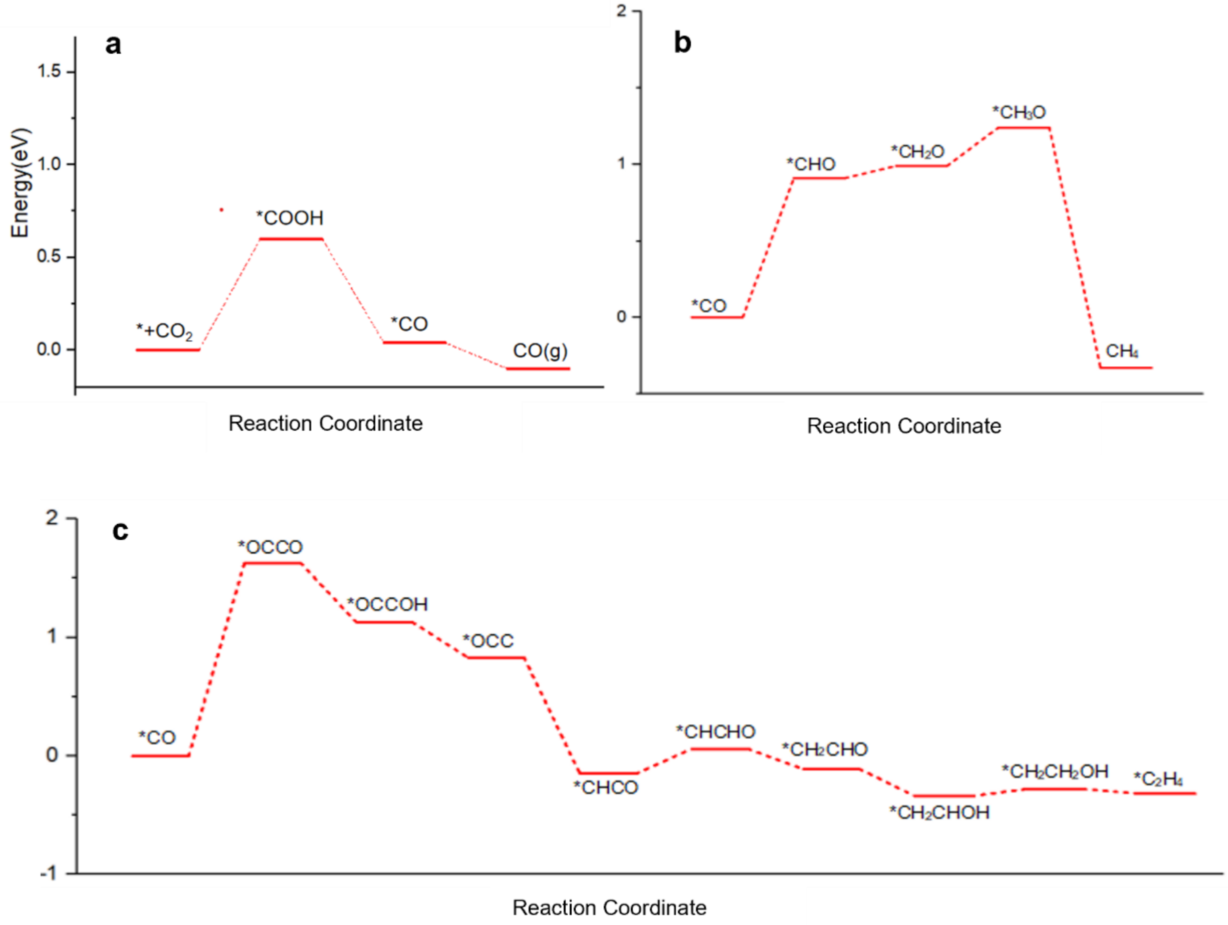

Figure S28. Catalytic pathways of CB[7]-Ni for the possible product of (a) CO, (b) CH_4, and (c) C_2H_4 in photocatalytic CO_2 reduction

In the reaction pathway for CH_4 formation, it shows that a high energy barrier of 1.24 eV is required to form the *CH_3O intermediate (Figure S28b). In the catalytic pathway for C_2H_4 formation, the most challenging step is C–C coupling, which has an even higher energy barrier of 1.63 eV (Figure S28c). This makes C–C coupling much less favorable. In the CO_2 to CO reaction pathway, the formation of *COOH intermediate shows an energy barrier of 0.62 eV, followed by the thermodynamically favorable CO desorption in subsequent steps (Figure S28a). The comparison of the energy barriers across the three catalytic pathways highlights the favorable pathway for CO_2-to-CO conversion, consistent with the high CO yield and selectivity observed for CB[7]-Ni in experimental performance results.

Table S1. Comparison of reported homogeneous photocatalysts for CO_2-to-CO conversion. (λ > 300 nm)

Catalyst	[catalyst]	Photosensitizer	CO yield rate (μmol h^{-1})	CO yield rate (μmol g^{-1}_{metal} h^{-1})	TON_{CO}/ TOF_{CO}	CO Selectivity (%)	QE (%)	Reaction conditions	Refs.
CB[7]-Ni	10 mg	$[Ru(bpy)_3]Cl_2$	72.1	*6.3 × 10^7	*TON 1844 *TOF 1.0 s^{-1}	97.9%	1.34%	λ = 420-780 nm, CH_3CN/TEOA/H_2O (4:1:1)	This work
CoCo-cryptate	0.025 μM	$[Ru(phen)_3](PF_6)_2$	0.21	1.4 × 10^7	TOF 0.47 s^{-1}	98%	0.04%	λ = 450 nm, H_2O/CH_3CN (1:4), TEOA (0.3 M)	[25]
$[Co^{II}(TPA)Cl]Cl$	5 μM	$Ir(ppy)_3$	1.73	1.46 × 10^6	TOF 0.013 s^{-1}	85%	n.r.	λ = 460 nm LED, TEA/CH_3CN (1:4)	[26]
$[Co(L-N_5)]^{2+}$	50 μM	$Ir(ppy)_3$	n.r.	/	TON 270	97%	n.r.	λ > 460 nm blue LED, CH_3CN/TEA (4:1)	[27]
Fe(0) porphyrin	2 μM	$Ir(ppy)_3$	n.r.	/	TON 40	92%	0.13%	λ > 420 nm, 0.36 M TEA	[28]
$[Co(dm-phen)]^{2+}$	1.5 mM	$[Ru(bpy)_3]Cl_2$	5.4	3050	/	17%	1.20%	λ = 410-750 nm, DMF/TEOA	[29]
Co-NTB	2 μM	$[Ru(phen)_3](PF_6)_2$	1.18	2.0 × 10^6	TON 1179; TOF 0.032	97%	0.22%	λ = 450 nm, H_2O/CH_3CN	[30]

					s^{-1}			(1:4), TEOA (0.3 M)	
[Ni(^{Pr}bimiq1)]$^{2+}$	0.2 μM	Ir(ppy)$_3$	3.0	2.4 × 10^7	TOF 3.9 s^{-1}	93%	0.01%	λ > 420 nm, in a 0.07 M TEA, CH_3CN	[31]
[$Fe_3(CO)_{12}$]	400 μM	[Ru(bpy)$_3$]Cl_2	~40	23065	TON 100	50%	5.20%	λ = 440 nm, NMP/TEOA (5:1)	[32]
[Co(qpy)(OH_2)$_2$]$^{2+}$	5 μM	[Ru(bpy)$_3$]Cl_2	27	4.5 × 10^7	TON 2660	98%	2.80%	λ = 460 nm LED, 0.1 M BIH, 0.5 M TEOA, CH_3CN	[33]
[Cu(qpy)]$^{2+}$	1 μM	[Ru(bpy)$_3$]Cl_2	10.4	6.5 × 10^7	TON 12400	97%	1.20%	BIH (0.1 M)/TEOA (15% v/v) in H_2O/ CH_3CN (3% v/v)	[34]

*[Reaction condition: 15 mg [Ru(bpy)$_3$]Cl_2, 12 mL mixed solvent (CH_3CN:TEOA:H_2O = 4:1:1), 0.02 μM CB[7]-Ni, reaction time 0.5 h, 300 W Xe lamp (λ > 420 nm)] The yield of CO is 0.83 μmol after half hour reaction.

Table S2. Comparison of reported heterogeneous photocatalysts for CO_2-to-CO conversion. (λ > 400 nm)

Catalyst	[catalyst]	Photosensitizer	CO yield rate ($\mu mol\ h^{-1}$)	CO yield rate ($\mu mol\ g^{-1}_{cat}\ h^{-1}$)	TON_{CO}/ TOF_{CO}	CO Selectivity (%)	QE (%)	Reaction conditions	Refs.
CB[7]-Ni	10 mg	$[Ru(bpy)_3]Cl_2$	72.1	*1.7×10^6	*TON 1844 *TOF 1.0 s^{-1}	97.9%	1.34%	λ = 420-780 nm, $CH_3CN/TEOA/H_2O$ (4:1:1)	This work
Co_3O_4 hexagonal platelets	10 mg	$[Ru(bpy)_3]Cl_2$	20.03	2003	/	77.1%	0.07%	CH_3CN /H_2O/TEOA (3:1:1), 10 mg Ru, 15 ˚C	[35]
g-C_3N_4/CdS	20 mg	/	4.69	234.6	/	73.4%	/	7 mL CH_3CN, 0.5 mL H_2O, 0.5 g TEOA, 4 μmol $[Co(bpy)_3]Cl_2$ (as co-catalyst)	[36]
Pd-CdS	15 mg	/	0.36	24	/	100%	/	Photocatalyst dispersed in 1 mL H_2O (as hole scavenger)	[37]
TiO_2/graphene MoS_2	/	/	0.46	92.3	/	97%	/	Photocatalyst dispersed in 4 mL H_2O, 40 ˚C	[38]
TiO_{2-x} {001}-{101}	40 mg	/	0.44	11	/	/	0.31%	H_2O vapor (as co-catalyst) in a continuous flow mode, 150 °C	[39]

TiO_2@50Cu	10 mg	/	0.27	27	/	68%	/	H_2O vapor (as co-catalyst)	[40]
UIO-66/C_3N_4	/	/	/	9.79	/	/	/	CH_3CN /TEOA (4:1)	[41]
MOF-525-Co<br>MOF-525-Zn	2 mg	/	0.40<br>0.22	200.6<br>111.7	/	40%	/	CH_3CN /TEOA (4:1)	[42]
CoP/CNT<br>CoP/rGO	1 mg	[Ru(bpy)$_3$]Cl_2	39.51<br>47.33	39510<br>47330	/<br>/	73.1%	/	CH_3CN /H_2O/TEOA (4:1:1.5), 7.5mg Ru, 25 ˚C, white LED	[43]
Co-Fe PBA colloidal cluster	1 mg	[Ru(bpy)$_3$]Cl_2	11.70	11700	/	77.5%	0.97%	CH_3CN /H_2O/TEOA (3:2:1), 10 umol Ru, 30 ˚C	[44]
CoO_x/MIL-101(Cr)	1 mg	[Ru(bpy)$_3$]Cl_2	28.70	28700	/	70.3%	/	CH_3CN /H_2O/TEOA (3:2:1),<br>10 μmol Ru, CO_2 (1 atm)	[45]
$Ni(OH)_2$ graphene nanosheets	1 mg	[Ru(bpy)$_3$]Cl_2	10.7	10725	TON 4.8	96%	1.03%	MeCN/H_2O/TEOA (3:2:1)	[46]

*[Reaction condition: 15 mg [Ru(bpy)$_3$]Cl_2, 12 mL mixed solvent (CH_3CN:TEOA:H_2O = 4:1:1), 0.02 μM CB[7]-Ni, reaction time 0.5 h, 300 W Xe lamp (λ > 420 nm)] The yield of CO is 0.83 μmol after half hour reaction.

Table S3. Gas product yields of CB[7]-Ni coupled with RhB for photocatalytic CO_2 reduction

CB[7]-Ni with RhB	CO	H_2	CO selectivity (%)
Gas product yields (μmol)	0.35	0.08	81.4%

Reaction condition: 10 mg CB[7]-Ni catalyst, 11.5 mg RhB (the same mole as the mole of 15 mg $[Ru(bpy)_3]Cl_2$ for comparison); 12 mL solution (MeCN:TEOA:H_2O= 4:1:1); 300 W Xe lamp with a cut-off filter for visible light (λ > 420 nm), reaction time 0.5 h.

Table S4. The percentage of Ni content in CB[7]-Ni and CB[7]-AMD-Ni determined by ICP-OES

Sample	Element Percentage (wt. %)
CB[7]-Ni	2.7
CB[7]-AMD-Ni	1.5

Table S5. Parameters of Ni K-edge EXAFS fitting for CB[7]-Ni, standard Ni foil and NiO. (S_0^2 = 0.864)

Sample	Shell	CN^a	R(Å)^b	σ^2(Å^2)^c	ΔE_0(eV)^d	R factor
CB[7]-Ni	Ni-O	6.3±0.6	2.048±0.008	0.0060±0.0011	-3.4±1.0	0.0108
Ni foil	Ni-Ni	12*	2.483±0.002	0.0061±0.0002	7.2±0.3	0.0019
NiO	Ni-O	6.0±0.3	2.082±0.005	0.0070±0.0010	0.9±0.9	0.0033
	Ni-Ni	12.3±0.6	2.950±0.012	0.0071±0.0005	2.6±1.8	

^{a}*CN*, coordination number; ^{b}*R*, the distance to the neighboring atom; $^c\sigma^2$, the Mean Square Relative Displacement (MSRD); $^d\Delta E_0$, inner potential correction; *R* factor indicates the goodness of the fit. S_0^2 was fixed to 0.864, according to the experimental EXAFS fit of Ni foil by fixing *CN* as the known crystallographic value.

*This value was fixed during EXAFS fitting, based on the known structure of Ni. Fitting range: 3.0 ≤ k (/Å) ≤ 14.0 and 1.0 ≤ R (Å) ≤ 3.0 (Ni foil); 3.0 ≤ k (/Å) ≤ 11.7 and 1.0 ≤ R (Å) ≤ 3.0 (NiO); 2.0 ≤ k (/Å) ≤ 12.3 and 1.0 ≤ R (Å) ≤ 2.5(CB[7]-Ni). A reasonable range of EXAFS fitting parameters: 0.700 < S_0^2 < 1.000; *CN* > 0; σ^2 > 0 Å^2; $|\Delta E_0|$ < 10 eV; *R* factor < 0.02.

Table S6. The characteristic main adsorption bands of CB[n] in various wavenumbers[47]

Assignment	CB[n] (cm^{-1})
ν(C=O)	1724
δ(CH_2)	1473
ω(CH_2)	1421
ν(C-N) + ν(C-C)	1375
ν(N-C-N) of glycoluril ring	1320
ν(N-C-N) of glycoluril ring	1230
ν(C-N-C)	1190
ν(C-C)	1026
ν(C-N)	968
Out-of-plane deformation of glycoluril ring	802

ν: stretching; δ: scissoring; ω: wagging

References

[1] J. Kim, I. S. Jung, S. Y. Kim, E. Lee, J. K. Kang, S. Sakamoto, K. Yamaguchi, K. Kim, *J. Am. Chem. Soc.* **2000**, *122*, 540–541.

[2] A. D. Becke, *J. Chem. Phys.* **1993**, *98*, 5648–5652.

[3] C. Lee, W Yang, R. G. Parr, *Physical review B* **1988**, 37, 785.

[4] A. D. McLean, G. S. Chandler, *J. Chem. Phys.* **1980**, *72*, 5639–5648.

[5] F. Weigend, R. Ahlrichs, *Physical Chemistry Chemical Physics* **2005**, *7*, 3297–3305.

[6] S. Grimme, *J. Comput. Chem.* **2006**, *27*, 1787–1799.

[7] T. Schwabe, S. Grimme, *Physical Chemistry Chemical Physics* **2007**, *9*, 3397–3406.

[8] E. Runge, E. K. U. Gross, *Physical review letters,* **1984***,* 52, 997.

[9] A. V. Marenich, C. J. Cramer, D. G. Truhlar, *Journal of Physical Chemistry B* **2009**, *113*, 6378–6396.

[10] Y. Yao, Y. Gao, L. Ye, H. Chen, L. Sun, *J. Energy Chem.* **2018**, *27*, 502–506.

[11] Y. Wang, X. Liu, X. Han, R. Godin, J. Chen, W. Zhou, C. Jiang, J. F. Thompson, K. B. Mustafa, S. A. Shevlin, J. R. Durrant, Z. Guo, J. Tang, *Nat. Commun.* **2020**, *11*, 2531.

[12] D. R. Chowdhury, L. Spiccia, S. S. Amritphale, A. Paul, A. Singh, *J. Mater. Chem. A* **2016**, *4*, 3655–3660.

[13] D. Sigwalt, P. Y. Zavalij, L. Isaacs, *Supramol. Chem.* **2016**, *28*, 825–834.

[14] J. Kim, I. S. Jung, S. Y. Kim, E. Lee, J. K. Kang, S. Sakamoto, K. Yamaguchi, K. Kim, *J. Am. Chem. Soc.* **2000**, *122*, 540–541.

[15] Y. Zhao, Z. Liu, *Chin. J. Chem.* **2018**, *36*, 455–460.

[16] P. G. Alsabeh, A. Rosas-Hernández, E. Barsch, H. Junge, R. Ludwig, M. Beller, *Catal. Sci. Technol.* **2016**, *6*, 3623–3630.

[17] A. McNally, B. Haffemayer, B. S. L. Collins, M. J. Gaunt, *Nature* **2014**, *510*, 129–133.

[18] S. J. Barrow, S. Kasera, M. J. Rowland, J. Del Barrio, O. A. Scherman, *Chem. Rev.* **2015**, *115*, 12320–12406.

[19] A. Wagner, K. H. Ly, N. Heidary, I. Szabó, T. Földes, K. I. Assaf, S. J. Barrow, K. Sokołowski, M. Al-Hada, N. Kornienko, M. F. Kuehnel, E. Rosta, I. Zebger, W. M. Nau, O. A. Scherman, E. Reisner, *ACS Catal.* **2020**, *10*, 751–761.

[20] X. Du, X. Liu, H. Su, X. Cheng, L. Li, H. Gu, X. Xing, D. Qiu, H. Hao, *Microchemical Journal* **2022**, *182*, 107942.

[21] N. J. Wheate, D. P. Buck, A. I. Day, J. G. Collins, *Dalton Trans.* **2006**, 451–458.

[22] X. Li, J. Yu, M. Jaroniec, *Chem. Soc. Rev.* **2016**, *45*, 2603–2636.

[23] J. A. Marsella, *Kirk-Othmer Encyclopedia of Chemical Technology*, **2000**, 1-9.

[24] S. K. Samanta, J. Quigley, B. Vinciguerra, V. Briken, L. Isaacs, *J. Am. Chem. Soc.* **2017**, *139*, 9066–9074.

[25] T. Ouyang, H.-J. Wang, H.-H. Huang, J.-W. Wang, S. Guo, W.-J. Liu, D.-C. Zhong, T.-B. Lu, *Angew. Chem.* **2018**, *130*, 16718–16723.

[26] S. L. F. Chan, T. L. Lam, C. Yang, S. C. Yan, N. M. Cheng, *Chem. Commun.* **2015**, *51*, 7799–7801.

[27] L. Chen, Z. Guo, X. G. Wei, C. Gallenkamp, J. Bonin, E. Anxolabéhère-Mallart, K. C. Lau, T. C. Lau, M. Robert, *J. Am. Chem. Soc.* **2015**, *137*, 10918–10921.

[28] J. Bonin, M. Robert, M. Routier, *J. Am. Chem. Soc.* **2014**, *136*, 16768–16771.

[29] R. Ziessel, J. Hawecker, J. -M Lehn, *Helv. Chim. Acta* **1986**, *69*, 1065–1084.

[30] T. Ouyang, C. Hou, J. W. Wang, W. J. Liu, D. C. Zhong, Z. F. Ke, T. B. Lu, *Inorg. Chem.* **2017**, *56*, 7307–7311.

[31] V. S. Thoi, N. Kornienko, C. G. Margarit, P. Yang, C. J. Chang, *J. Am. Chem. Soc.* **2013**, *135*, 14413–14424.

[32] P. G. Alsabeh, A. Rosas-Hernández, E. Barsch, H. Junge, R. Ludwig, M. Beller, *Catal. Sci. Technol.* **2016**, *6*, 3623–3630.

[33] Z. Guo, S. Cheng, C. Cometto, E. Anxolabéhère-Mallart, S. M. Ng, C. C. Ko, G. Liu, L. Chen, M. Robert, T. C. Lau, *J. Am. Chem. Soc.* **2016**, *138*, 9413–9416.

[34] Z. Guo, F. Yu, Y. Yang, C. F. Leung, S. M. Ng, C. C. Ko, C. Cometto, T. C. Lau, M. Robert, *ChemSusChem* **2017**, *10*, 4009–4013.

[35] C. Gao, Q. Meng, K. Zhao, H. Yin, D. Wang, J. Guo, S. Zhao, L. Chang, M. He, Q. Li, H. Zhao, X. Huang, Y. Gao, Z. Tang, *Adv. Mater.* **2016**, *28*, 6485–6490.

[36] N. N. Vu, S. Kaliaguine, T. O. Do, *ACS Appl. Energy Mater.* **2020**, *3*, 6422–6433.

[37] Q. Chen, S. Wu, S. Zhong, B. Gao, W. Wang, W. Mo, H. Lin, X. Wei, S. Bai, J. Chen, *J. Mater. Chem. A* **2020**, *8*, 21208–21218.

[38] H. Jung, K. M. Cho, K. H. Kim, H. W. Yoo, A. Al-Saggaf, I. Gereige, H. T. Jung, *ACS Sustain. Chem. Eng.* **2018**, *6*, 5718–5724.

[39] L. Liu, Y. Jiang, H. Zhao, J. Chen, J. Cheng, K. Yang, Y. Li, *ACS Catal.* **2016**, *6*, 1097–1108.

[40] M. Liu, L. Zheng, X. Bao, Z. Wang, P. Wang, Y. Liu, H. Cheng, Y. Dai, B. Huang, Z. Zheng, *Chemical Engineering Journal* **2021**, *405*, 126654.

[41] L. Shi, T. Wang, H. Zhang, K. Chang, J. Ye, *Adv. Funct. Mater.* **2015**, *25*, 5360–5367.

[42] H. Zhang, J. Wei, J. Dong, G. Liu, L. Shi, P. An, G. Zhao, J. Kong, X. Wang, X. Meng, J. Zhang, J. Ye, *Angew. Chem.* **2016**, *128*, 14522–14526.

[43] Z. C. Fu, R. C. Xu, J. T. Moore, F. Liang, X. C. Nie, C. Mi, J. Mo, Y. Xu, Q. Q. Xu, Z. Yang, Z. S. Lin, W. F. Fu, *Chem. Eur. J.* **2018**, *24*, 4273–4278.

[44] J. Nai, S. Wang, W. Xiong, D. Lou, Science Advances, **2019**, 5, eaax5095.

[45] Y. Ma, J. Du, Y. Fang, X. Wang, *ChemSusChem* **2021**, *14*, 946–951.

[46] K. Q. Lu, Y. H. Li, F. Zhang, M. Y. Qi, X. Chen, Z. R. Tang, Y. M. A. Yamada, M. Anpo, M. Conte, Y. J. Xu, *Nat. Commun.* **2020**, *11*, 5181.

[47] Z. Li, L. Li, D. Hu, C. Gao, J. Xiong, H. Jiang, W. Li, *J. Colloid Interface Sci.* **2019**, *539*, 400–413.
